# Potential role of lactylation in intrinsic immune pathways in lung cancer

**DOI:** 10.3389/fphar.2025.1533493

**Published:** 2025-03-17

**Authors:** Mengdie Huang, Ye Jin, Dandan Zhao, Xingren Liu

**Affiliations:** ^1^ Department of Pulmonary and Critical Care Medicine, Sichuan Provincial People’s Hospital, University of Electronic Science and Technology of China, Chengdu, China; ^2^ Department of Thoracic Surgery, Sichuan Provincial People’s Hospital, University of Electronic Science and Technology of China, Chengdu, China

**Keywords:** lactylation, cGAS-STING, TLR, RIG-I, lung cance

## Abstract

Lung cancer, one of the most lethal malignancies, has seen its therapeutic strategies become a focal point of significant scientific attention. Intrinsic immune signaling pathways play crucial roles in anti-tumor immunity but face clinical application challenges despite promising preclinical outcomes. Lactylation, an emerging research focus, may influences lung cancer progression by modulating the functions of histones and non-histone proteins. Recent findings have suggested that lactylation regulates key intrinsic immune molecules, including cGAS-STING, TLR, and RIG-I, thereby impacting interferon expression. However, the precise mechanisms by which lactylation governs intrinsic immune signaling in lung cancer remain unclear. This review presents a comprehensive and systematic analysis of the relationship between lactylation and intrinsic immune signaling pathways in lung cancer and emphasizes the innovative perspective of linking lactylation-mediated epigenetic modifications with immune regulation. By thoroughly examining current research findings, this review uncovers potential regulatory mechanisms and highlights the therapeutic implications of targeting lactylation in lung cancer. Future investigations into the intricate interactions between lactylation and intrinsic immunity are anticipated to unveil novel therapeutic targets and strategies, potentially improving patient survival outcomes.

## 1 Introduction

Lung cancer has become the leading cause of death in both males and females at a younger age (Bade and Dela Cruz, 2020). In addition, the economic burden of lung cancer is also increasing year by year, and it has become an urgent social and public health problem (Bade and Dela Cruz, 2020; Hirsch et al., 2017). Lung cancer is mainly categorized into two main types: non-small cell lung cancer (NSCLC) and small cell lung cancer (SCLC). Among them, NSCLC accounts for more than 80% of lung cancer cases, covering various subtypes such as adenocarcinoma, squamous carcinoma, and large cell carcinoma etc. NSCLC grows relatively slowly, but it is often diagnosed at an advanced stage, making it significantly more difficult to treat. On the other hand, SCLC is characterized by rapid growth, early metastasis, and high malignancy, and has already spread throughout the body at the time of diagnosis, resulting in poor therapeutic outcomes (Hirsch et al., 2017; de Sousa and Carvalho, 2018; Nooreldeen and Bach, 2021; Wang et al., 2020a).

The human body has a strong immune surveillance function that can recognize and remove potential tumor cells, thus preventing tumor formation. Innate immunity, as the body’s first solid line of antiviral and antitumor defense, recognizes pathogen-associated molecular patterns (PAMPs) and damage-associated molecular patterns (DAMPs) through pattern recognition receptors (PRRs), which in turn activate key signaling pathways such as cGAS-STING, TLR, and RIG-I. The activation of these pathways can trigger the release of interferon and the production of a series of cytokines, thus effectively promoting the establishment of anti-tumor immunity (Demaria et al., 2019; Taguchi and Mukai, 2019). However, although many preclinical studies have demonstrated that activation of intrinsic immune pathways has significant antitumor effects, agonists of intrinsic immune pathways have achieved only limited success in the clinical setting (Welander, 1987; Holicek et al., 2023; Yi et al., 2023). Therefore, an in-depth investigation of the specific mechanism of action of the intrinsic immune pathway in tumors is important for crossing the translational divide from preclinical to clinical settings.

Recent research has spotlighted lactate as a critical player in the tumor microenvironment, influencing tumor cell behavior through mechanisms such as signaling regulation, angiogenesis promotion, and immune suppression (Apostolova and Pearce, 2022; Liu et al., 2024; Wang et al., 2020b). For instance, PM2.5 exposure enhances glycolytic metabolism via DLAT transcription and translation, thereby facilitating NSCLC progression (Chen et al., 2022a). Conversely, hyperoxic environments inhibit lung cancer growth and invasiveness by suppressing the MYC/MCT1 axis, leading to intracellular lactate accumulation and acidification (Liu et al., 2023a). Identified as a novel post-translational modification (PTM), lactylation provides a mechanistic insight into how lactate exerts its regulatory functions in cancer biology. Histone lysine lactylation (Kla) and non-histone protein lactylation represent critical mechanisms by which lactate regulates gene expression and cellular functions (Chen et al., 2022b; Lv et al., 2023; Wang et al., 2022a; Yu et al., 2021). Unlike other PTMs such as acetylation or methylation, lactylation directly links cellular metabolism to epigenetic regulation, thus influencing immune signaling pathways and immune cell differentiation.

Notably, lactylation has been shown to regulate key intrinsic immune molecules (e.g., cGAS), and then affect intrinsic immunity. For example, AARS1/2-mediated lactylation of cGAS inhibits its activation of intrinsic immunity by impairing phase separation and DNA sensing, thus attenuating immune surveillance. Inhibition of lactylation via MCT1 blockade restores cGAS function, enhancing immune responses and suppressing viral replication (Li et al., 2024a). These results indicate that lactylation acts as a metabolic regulator, intricately linking cellular metabolism with immune signaling pathways. Despite the emerging evidence connecting lactylation to intrinsic immune regulation, the specific mechanisms underlying lactylation-mediated modulation of intrinsic immune signaling pathways in lung cancer remain largely unexplored.

Given the pivotal role of intrinsic immunity in anti-tumor defense and the potential of lactylation as an immunoregulatory mechanism, there is an urgent need to conduct a comprehensive understanding of their interactions. This review presents a systematic and in-depth analysis of the relationships between lactylation and intrinsic immune signaling pathways in lung cancer and emphasizes the novel perspective of linking lactylation-mediated epigenetic modifications with immune regulation. By synthesizing current research findings, this review aims to elucidate the potential mechanisms through which lactylation influences intrinsic immune signaling in lung cancer. We anticipate that this study will provide a robust theoretical foundation and identify novel therapeutic targets for lung cancer immunotherapy, paving the way for innovative treatment strategies and improved patient survival.

## 2 Lactylation and lung cancer

### 2.1 Biological mechanisms of lactylation

As a novel form of post-translational modification (PTM), lactylation was initially revealed with the help of mass spectrometry in 2019 by Zhao et al. (Zhang et al., 2019; Xu et al., 2024). They identified the lactylation occurring on histone lysine, named lysine lactylation (Kla), as an unprecedented chemical modification (Zhang et al., 2019; Xu et al., 2024). Since then, research in this field has continued to intensify, revealing that lactylation is not limited to histones and widespread in a wide range of non-histone proteins. The core of lactylation is that lactate molecules are covalently bonded to lysine residues of proteins, which can have a profound effect on the function and biological activity of proteins (Hu et al., 2024a). Lactylation has greatly broadened the boundaries of our knowledge about the role of lactate in cellular metabolism and epigenetic regulation, and opened up new research pathways for exploring its potential regulatory mechanisms in physiological and pathological processes.

**FIGURE 1:**
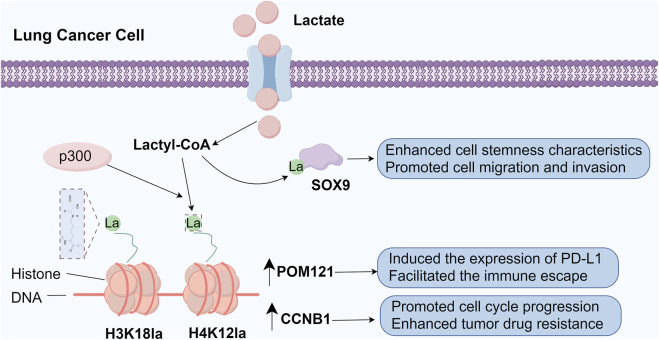
The role of histone and non-histone lactylation modifications in lung cancer (By figdraw). Lactylation of SOX9 promotes glycolysis and enhances stemness, migration, and invasion potential in tumor cells. Elevated lactylation at H4K12 significantly increases CCNB1 transcription, driving cell cycle progression and conferring resistance to pemetrexed. Upregulated lactylation at H3K18 directly activates POM121 transcription, facilitating MYC nuclear translocation, inducing PD-L1 expression, and ultimately promoting tumor immune evasion.

There is a complex mechanism of lactylation, involving both enzymatic and non-enzymatic pathways: (1) In the context of enzymatic reactions, protein lactylation is primarily catalyzed by specific enzymes. Histone acetyltransferases (HATs) play a central role in this process. Early studies identified p300 as the first protein confirmed to have lactylation writing capability. In HEK293T cells, overexpression of p300 led to a slight increase in histone Kla (lysine lactylation) levels, while silencing p300 in HCT116 and HEK293T cells decreased Kla levels, confirming p300 as a potential Kla writer enzyme (Xu et al., 2024; Zeng et al., 2023; Huang et al., 2018) (Figure 1). In addition, a 2022 study on myocardial infarction revealed that GCN5 significantly catalyzes H3K18la (histone H3 lysine 18 lactylation) modifications (Wang et al., 2022a). Both p300 and GCN5 belong to the HAT family, indicating that multiple members of this family might participate in lactylation modifications (Marmorstein and Zhou, 2014). A recent study identified a new lysine lactyltransferase, HB01, which regulates histone Kla both *in vivo* and *in vitro*, with a preference for catalyzing H3K9la (Niu et al., 2024). HB01 belongs to the MYST family and exhibits multifunctional histone acyltransferase activity, capable of catalyzing lactylation, acetylation, propionylation, butyrylation, crotonylation, and benzoylation (Niu et al., 2024). These reactions utilize lactyl-CoA as a substrate, through which lactate groups are covalently added to lysine residues via enzymatic catalysis. (2) In terms of non-enzymatic mechanisms, Galligan et al. proposed a passive modification process based on lactoyl glutathione (LGSH). This mechanism may modify proteins by non-specific lactylation through acyl transfer in an environment of high intracellular lactate concentration (Gaffney et al., 2020).

Lactylation erasers play a crucial role in regulating lactylation modifications. Histone deacetylases (HDACs), including HDAC1-3, HDAC8, and SIRT1-3, have been identified as histone lactylation erasers (Moreno-Yruela et al., 2022). *In vitro* studies have demonstrated the de-lactylase activity of HDAC1-3 and SIRT1-3. Overexpression and RNA interference experiments confirmed that HDAC1 and HDAC3 exhibit de-lactylase activity in cells (Moreno-Yruela et al., 2022). Notably, HDAC3 regulates histone lactylation at H4K5 and multiple sites (Gallinari et al., 2007; Xu et al., 2025). Additionally, SIRT3 shows the highest erasing activity at the H4K16la site compared to other human Sirtuins, suggesting site-specific regulation by different erasers (Fan. et al., 2023a).

Proteins recognizing lactylation modifications (readers) are key to the biological functions of lactylation. In 2024, a study on induced pluripotent stem cells (iPSCs) reprogramming reported for the first time that Brg1 is a reader of histone lactylation (Hu et al., 2024b). It was found that H3K18la modifications induced by Dux overexpression control the metabolic H3K18la-MET network and recruit p300 via the C-terminal domain, enhancing iPSC reprogramming efficiency. Further H3K18la immunoprecipitation experiments revealed that Brg1 is specifically recruited during reprogramming and is enriched at promoters of genes related to pluripotency and epithelial junctions, promoting the mesenchymal-to-epithelial transition (MET) process (Hu et al., 2024b).

The lysine sites targeted by lactylation exhibit some specificity across different proteins and biological systems. For example, studies have clearly identified lactylation sites on various histones, such as H3K18la and H4K12la. These modifications play crucial roles in the regulation of gene transcription (Li et al., 2024b; Wang et al., 2024a; Wei et al., 2023; Li et al., 2024c; Pan et al., 2022). In addition, the presence of lactylation has also been observed in enzymes (e.g., glycolytic enzymes ALDOA, PKM2) and signaling proteins (e.g., HMGB1), suggesting a broad regulatory role of lactylation in various biological processes (Feng et al., 2024; Wang et al., 2022b; Yang et al., 2022).

### 2.2 The central role of lactate metabolism in lung cancer pathogenesis, progression and treatment resistance

Lactate, traditionally perceived as a terminal metabolite of the glycolytic pathway, plays a pivotal role in the tumor microenvironment (TME) (Wang et al., 2020b). In recent years, numerous studies have focused on the phenomenon of metabolic reprogramming in tumor cells, especially the profound changes in lactate metabolism, revealing its central role in lung cancer initiation, malignant progression, and treatment resistance (Byun, 2023). The expression profiles of genes related to lactate metabolism have become key indicators for prognostic assessment and therapeutic strategy development in lung adenocarcinoma, among which the construction of the lactate signature score (LaSig) provides a powerful tool for predicting patient survival and response to immunotherapy (Chang et al., 2022; Shang et al., 2022). Clinically, it is important to dynamically monitor lactate dehydrogenase (LDH) levels for the prognosis of immunotherapy efficacy and the guidance of clinical decision-making in patients with NSCLC before and during the course of treatment (Vlachostergios et al., 2015).

Lactate metabolism is closely linked to immune evasion mechanisms within the tumor microenvironment. For example, deletion of the LKB1 gene triggers overexpression of the lactate transporter protein MCT4 and a significant increase in lactate secretion. This change not only promotes the polarization of M2-type macrophages, but also significantly suppresses T-cell function, which in turn weakens the anti-tumor immune response (Qian et al., 2023). In addition, lactate activates cancer-associated fibroblasts (CAF), and IL-8 released by CAF further recruits M2-type macrophages, exacerbating the immunosuppressive state in the tumor microenvironment (Gu et al., 2024a). In lung adenocarcinoma, high expression of NFATc2 upregulates the transcriptional activity of USP17. This enhances lactic acid production, promotes macrophage polarization toward the M2-type, and strengthens the immune evasion ability of the tumor. Intervention strategies targeting NFATc2 or USP17 can effectively reverse lactate-mediated immunosuppression and provide potential molecular targets for lung cancer therapy (Wang et al., 2024b).

Lactate metabolism is tightly intertwined with glycolytic processes, which together regulate the reprogramming of energy metabolism in tumor cells. etv4 promotes lactate production and activates the mTORC1 signaling pathway by upregulating hexokinase 1 (HK1) expression (Liu et al., 2023b). Meanwhile, enhanced stability of the deubiquitinating enzyme USP7 promoted c-Abl activation, thus upregulating HK2 expression and accelerating the glycolysis rate. High expression of PFKP in lung cancer regulate the level of glycolysis and promoted cellular proliferation (He et al., 2023); while downregulation of HERC5 lead to mitochondrial dysfunction, enhanced the Warburg effect, and elevated the invasive ability of tumor cells (Schneegans et al., 2024). In KRAS-mutated lung cancer, high expression of DRP1 regulated lactate utilization and redox homeostasis, providing support for tumor cell survival and proliferation (Hu et al., 2020).

Changes in lactate metabolism play a key role in lung cancer therapeutic resistance. For example, high expression of AKR1B10 in lung cancer brain metastases enhanced the Warburg effect and promoted glycolysis, leading to resistance to pemetrexed (PEM) (Duan et al., 2023). Lactate-induced metabolic reprogramming promoted cell cycle progression through upregulation of cell cycle protein B1 (CCNB1), which further enhanced tumor drug resistance (Duan et al., 2023). Persistent increase of LDH levels is closely associated with resistance to immune checkpoint inhibitor (ICI) therapy and poor prognosis in patients with advanced NSCLC, and serve as a biomarker for predicting the efficacy of immunotherapy. By targeting the lactate transporter protein MCT4 or blocking the lactate receptor GPR81, it is possible to reverse lactate-mediated immunosuppression and restore sensitivity to PD-1 blockade therapy (Qian et al., 2023; Chen et al., 2021). Formosanin C (FC) inhibits tumor progression by inhibiting the expression of MCT4 and CD147, blocking lactate efflux, and inducing mitochondrial dysfunction and oxidative stress (Li et al., 2023).

In summary, lactate metabolism plays a crucial role in the pathogenesis, progression, and therapeutic resistance of lung cancer, and it is expected to open up new strategies and targets for the treatment of lung cancer by precisely targeting the lactate metabolic pathway and its key regulatory mole.

### 2.3 Potential role of lactylation in lung cancer pathogenesis

Lactate, as an end product of the glycolytic pathway, has a function that is not limited to metabolic processes, but also participates in a novel post-translational modification, histone lactylation, as a donor for histone lactylation. This process involves the covalent addition of lactate groups to lysine residues of proteins, thereby profoundly affecting chromatin structure and gene expression patterns (Lv et al., 2023). In NSCLC cells, lactate exhibits fine regulation of metabolic pathways. It downregulates the expression of the key enzymes of glycolysis, HK-1 and PKM, while up-regulating the expression of the tricarboxylic acid cycle (TCA) enzymes, including SDHA and IDH3G. Alterations in the expression of these metabolic enzymes were closely associated with a significant elevation in the level of histone lactylation (Jiang et al., 2021; Chen et al., 2024; Guo et al., 2024; Zhang et al., 2024a). In addition, lactate induced lactylation of the transcription factor SOX9, which further promoted the glycolytic process and enhanced the stemness characteristics, migration and invasion potential of the cells (Yan et al., 2024). In NSCLC tissues, H3K18 lactylation levels were significantly upregulated and strongly associated with poor patient prognosis. This lactylation directly activated the transcription of POM121, which in turn enhanced the nuclear translocation of MYC, induced the expression of PD-L1, and ultimately facilitated the immune escape of the tumor. Notably, by inhibiting the glycolytic pathway, the level of H3K18 lactylation could be reduced, thereby enhancing the cytotoxicity of CD8^+^ T cells and effectively inhibiting tumor growth (Zhang et al., 2024a). In lung cancer brain metastases, high expression of AKR1B10 promoted glycolysis and lactate production, leading to a significant increase in the level of H4K12 lactylation. This change upregulated CCNB1 transcription, promoted cell cycle progression, and enhanced the resistance of tumor cells to pemetrexed (Duan et al., 2023). In addition to histones, lactylations are widely present on non-histone proteins, with profound effects on their stability and function. For example, lactic acid increases the stability of IGF1R by enhancing its lactylation, thereby promoting glycolysis and tumor cell proliferation (Zhang et al., 2024b). In addition, lactated APOC2 promoted extracellular lipolysis, a change that has been linked to the emergence of immunotherapy resistance (Chen et al., 2024). In addition, BZW2 accelerated the malignant progression of lung adenocarcinoma by promoting glycolysis-mediated lactate production and lactylation of IDH3G (Wang et al., 2023a) (Figure 1)

In view of the important role of lactylation in lung cancer pathogenesis, inhibition of histone lactylation has become a new strategy for lung cancer treatment. By targeting PKM2, the natural product Fargesin (FGS) effectively inhibit glycolysis and H3 histone lactylation, and significantly suppress the tumorigenesis of NSCLC. Meanwhile, inhibition of LDHA or LDHB to reduce the level of histone lactylation can enhance the function of CD8^+^ T cells, thereby suppressing immune escape from tumors (Guo et al., 2024). In addition, antibodies against lactated APOC2-K70 can enhance the efficacy of anti-PD-1 therapies, thereby overcoming immunotherapy resistance (Chen et al., 2024). In summary, lactate regulates gene expression through histone lactylation, profoundly affecting cell metabolism, proliferation and immune escape processes. Targeting lactylation and its related regulatory molecules provides a novel strategy for the treatment of lung cancer.

## 3 Intrinsic immune signaling pathway and lung cancer

### 3.1 Mechanism of cGAS-STING signaling pathway in lung cancer

STING (Stimulator of Interferon Genes), a transmembrane protein localized in the endoplasmic reticulum membrane, plays a central regulatory role in cytoplasmic DNA recognition and type I interferon (IFN) induction. When double-stranded DNA (dsDNA) is present in the cytoplasm, cyclic GMP-AMP synthase (cGAS) specifically recognizes and binds to these dsDNA molecules, which catalyzes the synthesis of the second messenger molecule, cGAMP. It serves as a key signaling molecule that activates STING proteins and triggers downstream signaling cascades, including the recruitment and activation of TANK-binding kinase 1 (TBK1) and interferon regulatory factor 3 (IRF3). This cascade of events ultimately induces the expression of type I interferons and pro-inflammatory cytokines, which initiate and regulate the innate immune response (Hopfner and Hornung, 2020; Chin et al., 2023; Ivashkiv and Donlin, 2014; Zhang et al., 2020).

It has been reported that activation of the cGAS-STING pathway exhibits significant anti-tumor effects in lung cancer. Specifically, TET2 effectively inhibit the proliferation and metastatic ability of lung adenocarcinoma cells by positively regulating the cGAS-STING pathway (Cheng et al., 2023). On the other hand, overexpression of ESYT3 significantly enhance the effect of radioimmunotherapy and inhibited lung adenocarcinoma growth through activation of the STING pathway (Luo et al., 2024). Knockdown of Flotillin-1 likewise activated STING signaling and enhanced the effect of radiotherapy (Wang et al., 2023b). In addition, GPR162, a novel tumor suppressor and radiosensitizer, effectively inhibits tumor progression by activating the STING-dependent DNA damage repair pathway (Long et al., 2023). Notably, tetrandrine (TET) activate the STING/TBK1/IRF3 signaling pathway by inducing DNA damage, which in turn enhances the effect of anti-PD-1 immunotherapy (Tan et al., 2024). This finding provides new strategies and ideas for immunotherapy of lung cancer. However, impaired function of the STING pathway may lead to immune escape and therapeutic resistance. In KEAP1-mutated NSCLC, EMSY accumulation inhibits type I interferon response, but promotes immune escape from cancer (Marzio et al., 2022). Amplification of the MET gene inhibits STING-mediated immunogenicity by up-regulating the expression of CD73 and reduces the response of EGFR-mutant lung cancer to immunotherapy (Yoshida et al., 2022).

Activation of the STING pathway promotes immune cell recruitment and activation, and profoundly alters the tumor immune microenvironment. For example, NR1D1 significantly increase the number of macrophages, dendritic cells and CD8^+^ T cells infiltrating in tumor tissues through activation of the cGAS-STING signaling pathway, thereby enhancing the anti-tumor immune effect and inhibiting breast cancer lung metastasis (Ka et al., 2023). Meanwhile, activation of the STING pathway also inhibits the expression of immunosuppressive factors and reverses the phenomenon of tumor immune escape. The interaction between LncRNA NEAT1 and DNMT1 inhibits the expression of p53, cGAS, and STING in lung cancer through epigenetic mechanisms, promoting the malignant phenotype of cancer cells and suppressing the infiltration of cytotoxic T cells. Inhibition of NEAT1 or DNMT1 expression restores STING pathway function, which enhances the anti-tumor immune response by promoting cytotoxic T cell infiltration (Ma et al., 2020). Rocaglamide (RocA), a small molecule compound, activates the cGAS-STING signaling pathway and promotes the infiltration and antitumor activity of natural killer (NK) cells in NSCLC (Yan et al., 2022). In addition to RocA and powdery mildew alkaloids, manganese ions (Mn^2+^) have been found to be important cofactors of the cGAS-STING pathway, which can enhance anti-tumor immune responses and improve the efficacy of clinical immunotherapy (Lv et al., 2020). Silencing of SAMHD1 induces macrophage polarization towards the M1-type and promotes infiltration of CD8^+^ T-cells through activation of IFI16-STING pathway. thereby enhancing the anti-tumor immune effect in lung adenocarcinoma (Li et al., 2022a).

**FIGURE 2:**
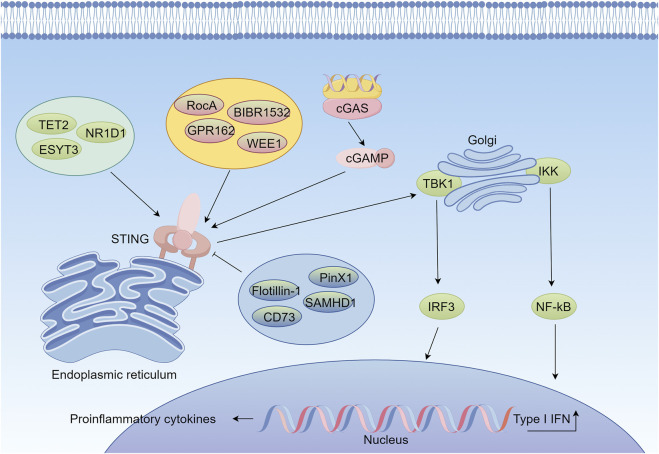
Mechanism of the cGAS-STING Signaling Pathway in Lung Cancer Immunotherapy (By figdraw). When cytosolic double-stranded DNA (dsDNA) is recognized by cyclic GMP-AMP synthase (cGAS), cGAS catalyzes the production of the second messenger molecule, cyclic GMP-AMP (cGAMP). cGAMP activates the STING protein located on the endoplasmic reticulum membrane, which subsequently recruits and phosphorylates the kinase TBK1 and the transcription factor IRF3. Phosphorylated IRF3, together with NF-κB, translocates into the nucleus, initiating the expression of type I interferons (IFNs) and pro-inflammatory cytokines, thereby activating the innate immune response. The activation of this pathway is regulated by multiple factors. Positive regulators include TET2, ESYT3, and NR1D1, which enhance STING signaling and activate the pathway. Small molecules such as GPR162, Rocaglamide (RocA), WEE1, and BIBR1532 directly enhance the function of the cGAS-STING pathway. Conversely, factors such as Flotillin-1, CD73, pINx1, and SAMHD1 inhibit the cGAS-STING pathway.

STING pathway has emerged as a promising strategy for combination therapy in lung cancer. Radiation therapy, a commonly used tumor treatment, can activate the STING pathway by inducing DNA damage, which in turn enhances anti-tumor immunity. Silencing PinX1 gene can significantly enhance the sensitivity and anti-tumor immune effect of NSCLC to radiotherapy by activating the cGAS-STING pathway (Qiu et al., 2024). BIBR1532 combined with radiotherapy induces NSCLC cells to undergo iron death while activating the cGAS-STING pathway, facilitating the occurrence and development of anti-tumor immunity (Bao et al., 2024). On the basis of STING pathway activation, the combination of radiotherapy and immunotherapy demonstrate significant synergistic effects. It was found that cryoablation therapy triggered type I interferon-dependent anti-tumor immune effects, thereby enhancing the efficacy of lung cancer immunotherapy (Gu et al., 2024b). Besides, radiotherapy combined with PD-L1 deletion and autophagy inhibition strategies significantly enhanced the antitumor effects in lung cancer through cGAS-STING-mediated T-cell activation mechanisms (Zhao et al., 2022a). WEE1 inhibitors activated STING and STAT1 pathways and enhanced antitumor immune responses to PD-L1 blockade therapies in SCLC (Taniguchi et al., 2022). ATR inhibitors in combination with radiotherapy enhanced the antitumor immune responses to PD-L1 blockade therapies by activating the STING-interferon signaling pathway, enhances the immunogenicity of SCLC and thus promotes anti-tumor immunity (Taniguchi et al., 2024). Moreover, inhibition of negative regulators of the STING pathway is also an effective strategy to indirectly activate STING signaling. By inhibiting the expression of molecules such as EMSY, CD73, and MET, the function of the STING pathway can be restored, thereby enhancing the effect of immunotherapy (Marzio et al., 2022; Yoshida et al., 2022; An et al., 2024; Jacoberger-Foissac et al., 2023). Metformin, a commonly used hypoglycemic agent, inhibits ubiquitination modification of STING through an AXIN1-dependent mechanism and then enhances the antitumor efficacy of STK11-mutant lung cancer against PD-1 inhibitors (Wang et al., 2022c) (Figure 2).

Although the importance of the STING pathway in lung cancer has been widely recognized, its clinical application still faces some challenges. For example, the side effects and dose modulation of STING agonists need further in-depth study. In addition, the complexity and heterogeneity of the tumor microenvironment significantly affects the activation of the STING pathway. Therefore, in future studies, it is necessary to further reveal the specific mechanism of STING pathway in lung cancer and explore safer and more effective STING agonists and combination therapies, with the aim of providing more precise and effective immunotherapy strategies for lung cancer patients.

### 3.2 Functions and regulatory mechanisms of TLR signaling pathway in lung cancer

Toll-like receptors (TLRs), as key components of the innate immune system, exhibit crucial roles in lung cancer development, progression, and immunoregulation. Based on their subcellular localization, TLRs have been classified into two major groups: membrane-localized TLRs 1, 2, 4, 5, 6, and 10 that are mainly responsible for recognizing microbial membrane components, and endosomal-localized TLRs 3, 7, 8, and 9, which mainly recognize microbial nucleic acids. Activation of TLRs induces downstream nuclear factor-κB (NF-κB) via MyD88-dependent or TRIF-dependent signaling pathways and interferon regulatory factors (IRFs) activation, which in turn triggers inflammatory responses and immune regulatory processes (Chakraborty et al., 2023; Hoden et al., 2022; Lim and Staudt, 2013; Zhou et al., 2024).

**FIGURE 3:**
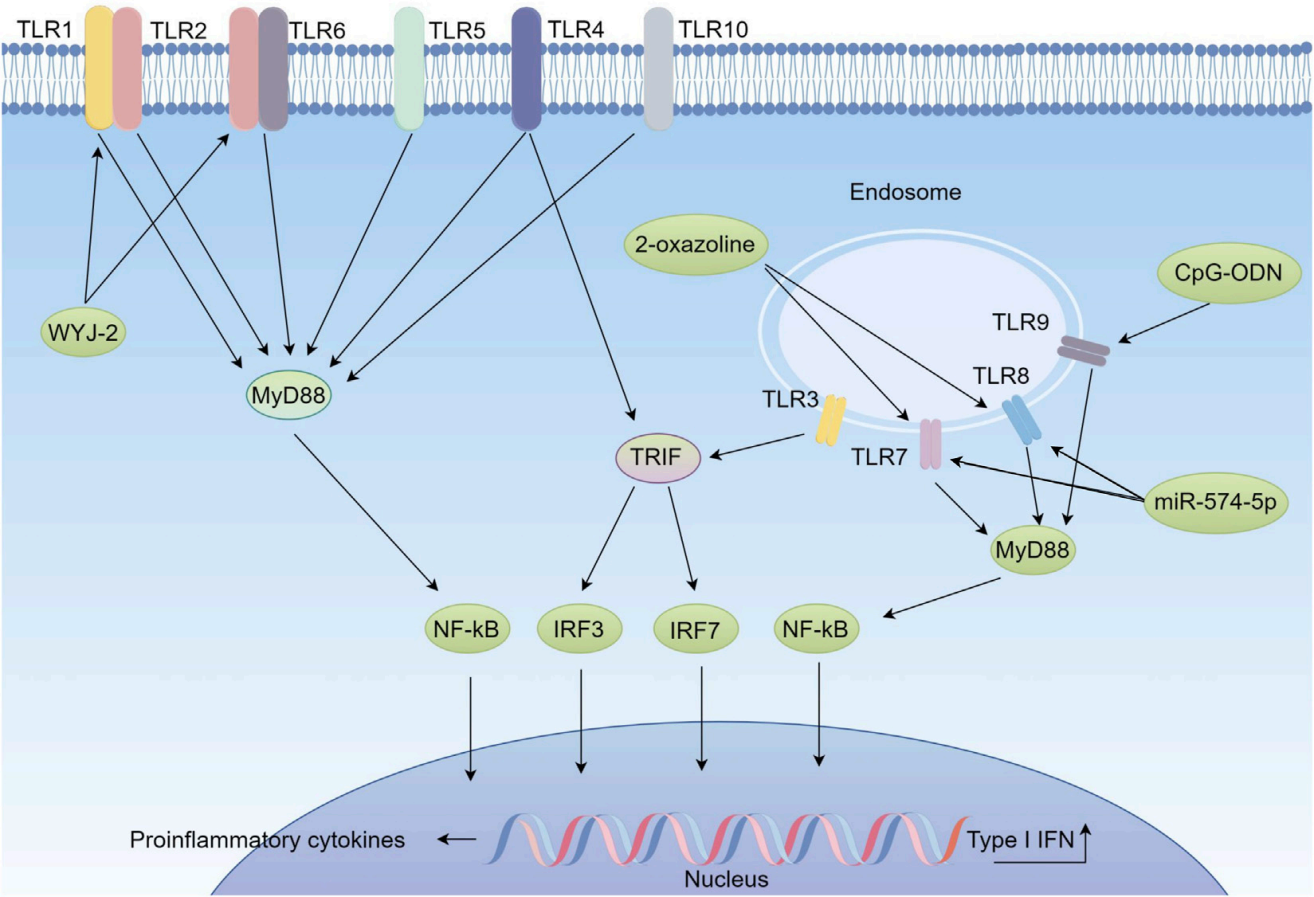
Function and Regulatory Mechanisms of the TLR Signaling Pathway in Lung Cancer (By figdraw). According to their subcellular localization, Toll-like receptors (TLRs) are mainly divided into two categories: membrane surface TLRs (including TLR1, TLR2, TLR4, TLR5, TLR6, and TLR10) and endosomal TLRs (including TLR3, TLR7, TLR8, and TLR9). Upon activation, TLRs transmit signals through two core pathways. The first is the MyD88-dependent pathway, utilized by TLR2/1, TLR2/6, TLR4, and TLR7/8. This pathway involves the adaptor protein MyD88, which activates nuclear factor κB (NF-κB) and drives the release of pro-inflammatory cytokines. The second is the TRIF-dependent pathway, used by TLR3 and TLR4. It involves the adaptor protein TRIF, which activates interferon regulatory factors IRF3/7, leading to the production of type I interferons (IFN-α/β). MicroRNA-574-5p derived from extracellular vesicles regulates the biosynthesis of prostaglandin E2 by activating TLR7/8, thereby influencing intercellular communication in lung cancer. This highlights the intricate role of TLR signaling in modulating the tumor microenvironment and cancer progression. Therapeutic strategies targeting the TLR pathway show significant potential. TLR agonists such as the TLR2/1 agonist WYJ-2 can inhibit NSCLC proliferation by inducing pyroptosis. Additionally, high-efficiency nanoparticle formulations of the TLR7/8 agonist Resiquimod, such as poly (2-oxazoline) carriers, significantly prolong survival and activate CD8^+^ T cell immune responses in chemotherapy-resistant lung adenocarcinoma models. TLR9 agonists, such as CpG-ODN delivered via nanoparticles, effectively promote tumor regression and enhance immune responses.

In lung cancer cells and immune cells in the tumor microenvironment, the abnormal expression patterns of TLRs are closely associated with multiple aspects of lung cancer, including tumorigenesis, progression, inflammatory response and immune cell infiltration. Specifically, TLR2 forms heterodimers with its co-receptors TLR1 or TLR6 and is involved in the regulation of the innate immune response (Farhat et al., 2008). Activation of TLR2 promotes migration, invasion and colony formation in lung cancer (Gergen et al., 2020). Notably, silent information regulator 2 (SIRT2) is secreted by macrophages upon TLR2 or TLR4 activation, further promoting the metastatic process of lung cancer (Wu et al., 2023). Furthermore, TLR2, 4 and 9 regulate the promotion of K-ras-driven lung cancer by chronic obstructive pulmonary disease (COPD)-like airway inflammation through activation of the MyD88/NF-κB pathway in the airway epithelium (Velasco et al., 2023).The high expression of TLR4 in lung cancer tissues is positively correlated with the degree of tumor malignancy, and its activation not only promotes proliferation and migration of lung cancer cells, but also induces immune escape mechanisms (Wang et al., 2017; Fu et al., 2013). Gram-negative bacterial infection significantly enhances the metastatic potential of NSCLC through activation of host TLR4 (Sun et al., 2018). In addition, activation of TLR4 upregulates the expression of programmed death ligand 1 (PD-L1), which in turn inhibits the anti-tumor activity of T cells and promotes immune escape (Kang et al., 2020). In the Lewis lung cancer mouse model, TLR4 also mediated the development of tumor-associated fatigue, a process independent of the activation status of macrophages and microglia (Vichaya et al., 2020). High levels of TLR7 gene expression are associated with poor clinical prognosis in patients with advanced NSCLC receiving immunotherapy (Baglivo et al., 2021). MicroRNA-574-5p of extracellular vesicle origin regulates prostaglandin E2 biosynthesis through activation of TLR7/8, which in turn affects the communication process between lung cancer cells (Donzelli et al., 2021).TLR pathway has emerged as a promising therapeutic strategy for lung cancer. The novel TLR2/1 agonist WYJ-2 inhibits the proliferation of NSCLC by promoting the formation of TLR2/1 heterodimers, activating the NF-κB signaling pathway, and inducing the focal death mechanism (Wang et al., 2024c). In PTEN-deficient squamous cell carcinoma of the lung, tumors are resistant to anti-PD-1 therapy and are accompanied by a high degree of regulatory T cell (Treg) infiltration and an immunosuppressive microenvironment. Treatment with TLR agonists in combination with anti-Transforming Growth Factor β (TGFβ) antibodies reverses this immunosuppressive state, leading to tumor rejection and the formation of immune memory (Exposito et al., 2023). In addition, fatty acid receptor 2 (FFAR2) signaling antagonizes TLR2- and TLR3-induced lung cancer progression by inhibiting the process of cyclic adenosine monophosphate (cAMP)-adenylate-activated protein kinase (AMPK)-transforming growth factor β-activated kinase 1 (TAK1)-NF-κB activation (Kim et al., 2023). TLR7/8 agonist Resiquimod demonstrated significant tumor suppression in a metastatic model of lung adenocarcinoma, and a high-capacity poly (2-oxazoline) formulation of Resiquimod prolonged survival and mobilized anti-tumor CD8^+^ immunoreactivity in a chemotherapy-insensitive model of metastatic lung adenocarcinoma (Vinod et al., 2020). TLR9 agonists, such as cytosine-guanine oligodeoxynucleotide (CpG-ODN), have been shown in preclinical and clinical trials to demonstrate potential as anticancer drugs (Zhang et al., 2021). Nanoparticle-conjugated TLR9 agonists delivered via the lungs were effective in treating metastatic lung cancer, promoting tumor regression and activating immune responses (Perry et al., 2020). In summary, the TLR pathway plays a complex and critical role in the development and progression of lung cancer. An in-depth investigation of the mechanism of action of the TLR pathway will provide an important basis for the development of new therapeutic strategies and the improvement of the prognosis of lung cancer patients (Figure 3).

### 3.3 Functions and regulatory mechanisms of RIG-I signaling pathway in lung cancer

Retinoic Acid-Inducible Gene I (RIG-I)-like Receptor Pathway (RLR pathway) is a core component of the innate immune response, which is mainly responsible for the recognition of viral RNA and the activation of antiviral immune response (Schweibenz et al., 2023; Thoresen et al., 2021; Xu et al., 2018). In recent years, more and more studies have shown that the RIG-I pathway plays a crucial role in the occurrence, progression and treatment of lung cancer (Jiang et al., 2023; Zheng et al., 2023). In lung cancer tissues and cell lines, the expression of RIG-I tends to be downregulated, and this change is closely related to the poor prognosis of lung cancer. As a key component of the RIG-I pathway, retinoic acid-induced gene G (Rig-G) is also frequently suppressed in lung cancer. Overexpression of Rig-G significantly inhibits lung cancer cell growth and migration and is accompanied by attenuated epithelial-mesenchymal transition (EMT). This process is closely related to the activation of the p53 signaling pathway, suggesting that the RIG-I pathway may inhibit lung cancer progression by regulating core tumor suppressors such as p53 (Sun et al., 2020). It was found that CHI3L1 could regulate the expression of immune checkpoint molecules such as PD-L1, PD-L2, and PD-1 to promote tumor progression. In contrast, activation of the RIG-I pathway can inhibit the expression of CHI3L1, which in turn downregulates molecules such as PD-L1, thereby enhancing anti-tumor immune responses (Ma et al., 2021). The downregulation of cyclic RNA circNDUFB2 expression in NSCLC tissues was negatively correlated with the malignant features of the tumors. circNDUFB2 acts as a scaffold that promotes the interaction of TRIM25 with IGF2BP, facilitating the ubiquitination and degradation of IGF2BP, thereby inhibiting tumor development. Moreover, circNDUFB2 can also be recognized by RIG-I, activate the RIG-I-MAVS signaling cascade, recruit immune cells into the tumor microenvironment, and further exert anti-tumor effects (Li et al., 2021).

Activation of the RIG-I pathway induces the production of type I interferon and various inflammatory factors, thereby promoting the recruitment and activation of immune cells. For example, application of RIG-I agonists enhances the apoptotic response of lung cancer cells to Fas ligand-induced apoptosis. The expression of Fas on the surface of lung cancer cells was upregulated after treatment with ionizing radiation (IR), at which time the use of RIG-I agonists was able to further enhance the sensitivity of cells to Fas ligand and induce significant apoptosis (Sato et al., 2020). hMENA11a, an isoform of actin cytoskeletal regulatory proteins, whose downregulation would be supported by RIG-I-mediated interferon-I signaling to maintain the tumor PD- L1 high expression activates the paracrine loop between tumor cells and macrophages, and promotes EMT. This mechanism may contribute to lung cancer resistance to immune checkpoint blockade (ICB) therapy (Trono et al., 2023). The efficacy of kinase inhibitors in the treatment of cancer is limited by resistance. Activation of the RIG-I pathway during kinase inhibitor therapy was found to significantly reduce tumor size by inducing inflammatory and pro-apoptotic responses that synergize with kinase inhibitors to reduce depleted CD8^+^ T cells (Bragelmann et al., 2021). In EGFR-mutated lung adenocarcinomas, defects in ARID1A correlate with adverse responses to immune checkpoint inhibitors (ICIs). Knockdown of ARID1A results in activation of the EGFR/PI3K/Akt/mTOR pathway and inhibition of autophagy, attenuates the inhibition of the RIG-I pathway activity, enhances the production of type I interferons, and reverses the resistance to ICIs (Sun et al., 2022).

**FIGURE 4:**
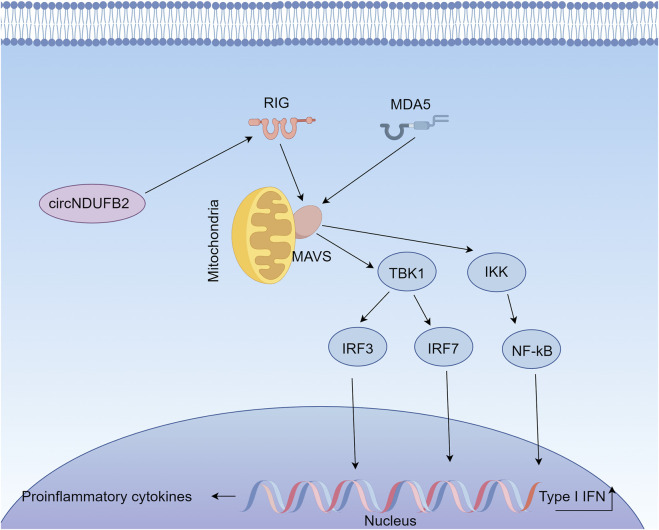
Function and Regulatory Mechanisms of the RIG-I Signaling Pathway in Lung Cancer (By figdraw). Retinoic acid-inducible gene I (RIG-I) and its homolog MDA5 recognize viral RNA or endogenous abnormal RNA,Upon activation, MAVS phosphorylates interferon regulatory factors IRF3/IRF7 and nuclear factor κB (NF-κB) through TBK1 (TANK-binding kinase 1) and the IKK (IκB kinase) complex, respectively. Once phosphorylated, IRF3/7 translocates into the nucleus to induce the transcription of type I interferons (IFN-α/β), while NF-κB promotes the release of pro-inflammatory cytokines. In lung cancer, abnormal regulation of this pathway is closely associated with tumor malignancy and the response to immunotherapy. Specifically, circNDUFB2 can act as a ligand for RIG-I, activating the RIG-I-MAVS signaling axis, enhancing immune cell infiltration, and exerting anti-tumor effects.

In summary, as a key component of the innate immune response, the RIG-I pathway plays an important role in the occurrence, progression and treatment of lung cancer. By activating the RIG-I pathway, the proliferation and metastasis of tumor cells can be inhibited, the tumor immune microenvironment can be improved, and the anti-tumor immune response can be enhanced, which provides new strategies and directions for the treatment of lung cancer (Figure 4).

## 4 New perspectives of lactylation regulating intrinsic immune interferon signaling

Epigenetic modifications are crucial mechanisms for regulating gene expression and cellular functions, with lactylation, acetylation, and methylation of the three major forms (Handy et al., 2011). These modifications differ significantly in their mechanisms, functions, and regulatory modes, and they play key roles in the tumor immune microenvironment and immune evasion (Handy et al., 2011; Garcia-Martinez et al., 2021).

Acetylation involves the addition of an acetyl group to lysine residues on histones or non-histone proteins, which relaxes chromatin structure and promotes gene transcription (Li et al., 2017). It also plays a vital role in tumor immune evasion. For example, SIRT1 deacetylates IRF3/IRF7, activating type I interferon (IFN-I) signaling and enhancing antiviral immunity. In contrast, SIRT1 deficiency leads to hyperacetylation of IRF3/IRF7, suppressing innate immunity and increasing susceptibility to viral infections (Author Anonymous, 2022). MYC downregulates H3K27 acetylation and JAK2 expression, impairing the IFN-γ signaling pathway and rendering lung cancer cells resistant to PD1/PD-L1 therapy (Alburquerque-Bejar et al., 2023). HBV upregulates HAT1, increasing H4K5/H4K12 acetylation, which inhibits the cGAS-STING-IFN-I pathway, facilitating innate immune evasion (Zhao et al., 2023). These findings demonstrate that acetylation plays a crucial role in regulating innate immunity and IFN signaling, making it a key mechanism of tumor and viral immune evasion.

Methylation involves the addition of a methyl group to lysine or arginine residues on histones or to cytosine residues on DNA, thereby regulating gene expression. Methylation can either activate or repress gene expression, depending on the modification site (Miller and Grant, 2013). For instance, targeting PRMT9-mediated arginine methylation inhibits the maintenance of cancer stem cells and enhances antitumor immunity by activating cGAS-mediated IFN-I responses. PRMT9 inhibition can also synergize with anti-PD-1 therapy, presenting a potential anticancer strategy (Dong et al., 2024). PRMT5 methylates IFN-γ inducible protein 16 (IFI16) and suppresses NLRC5 transcription, inhibiting the cGAS-STING and MHCI pathways in melanoma, thereby weakening IFN signaling and antitumor immune responses. PRMT5 inhibitors can restore IFN and chemokine production and enhance melanoma sensitivity to immune checkpoint inhibitors (Kim et al., 2020). Therefore, methylation plays a pivotal role in regulating innate immunity and IFN signaling, contributing to tumor immune evasion and antitumor immunity.

Lactylation, an emerging post-translational modification of lysine, has demonstrated a central role in regulating host gene expression networks in recent years (Chen et al., 2022b; Lv et al., 2023; Fan et al., 2023b; Li et al., 2022b; Xie et al., 2022). In recent studies, it has been found that lactylation can affect interferon production by regulating the expression of key molecules in the intrinsic immune signaling pathway. In systemic lupus erythematosus (SLE), cytosolic mtDNA stimulation triggers metabolic reprogramming in macrophages and dendritic cells, leading to a significant increase in lactate production. This metabolic byproduct not only fuels inflammatory responses but also specifically modifies the cGAS protein through lactylation. This post-translational modification alters the conformation of cGAS, enhancing its stability and preventing its interaction with the E3 ubiquitin ligase MARCHF5, thereby inhibiting ubiquitin-mediated proteasomal degradation. Stabilized cGAS continuously activates the STING signaling axis, amplifying the type I interferon (IFN-I) response and forming a positive feedback loop of autoimmune inflammation (Zhang et al., 2024c).

Notably, lactylation demonstrates significant heterogeneity in different pathological contexts. For example, during porcine reproductive and respiratory syndrome virus (PRRSV) infection, the virus induces lactylation at the H3K18 site of the host heat shock protein HSPA6, enhancing its stability and creating a novel interaction interface with IKKε kinase. This interaction competitively inhibits the recruitment of TRAF3 to the signaling complex, ultimately blocking IRF3 phosphorylation and NF-κB nuclear translocation. As a result, IFN-β production is suppressed, facilitating viral immune evasion (Pang et al., 2024). Further research has revealed that lactylation serves as a crucial bridge between immune metabolism and epigenetic regulation. The TLR signaling adapter BCAP activates the PI3K-AKT-mTOR pathway to drive glycolytic metabolism, generating lactate that acts as a substrate for histone modification. This mediates specific enrichment at the promoter regions of repair-related genes (e.g., Arg1, Klf4). This epigenetic remodeling alters chromatin accessibility, promoting the transition of macrophages from a pro-inflammatory M1 phenotype to a reparative M2 phenotype. In the absence of BCAP, the disruption of the metabolism-epigenetics axis leads to decreased lactate production and reduced histone lactylation levels, hindering tissue repair processes. However, supplementation with exogenous lactate can reverse this defect, confirming the central role of lactylation in cell fate determination (Irizarry-Caro et al., 2020). In tumor immune microenvironment regulation, a colorectal cancer liver metastasis (CRLM) model reveals a more complex lactylation network: Lactate accumulation promoted by intratumoral microbiota (e.g., *Escherichia coli*) induces lactylation at the K852 site of RIG-I, altering the conformation of its RNA recognition domain and blocking the assembly of the MAVS signaling complex. This suppresses NF-κB and IRF3 signaling pathways. Concurrently, lactylation of the NLRP3 inflammasome inhibits caspase-1 activation, reducing IL-1β and IL-18 secretion. This drives macrophage M2 polarization, enhances Treg immunosuppressive function, and inhibits CD8^+^ T cell cytotoxicity, constructing a multi-layered immune evasion system (Gu et al., 2024c).

These findings unveil the bidirectional regulatory characteristics of lactylation in maintaining immune homeostasis, pathogen defense, and tumor progression and lay a theoretical basis for precision therapeutic strategies targeting the metabolism-epigenetics interaction network.

## 5 Targeting lactylation and its potential in lung cancer immunotherapy

Lactylation, a post-translational modification driven by lactate, plays a crucial role in tumor metabolism and the immune microenvironment (Sui et al., 2025). It potentially influences type I interferon responses and inflammatory reactions by regulating the cGAS-STING, TLR, and RIG-I signaling pathways. Drug development targeting lactylation primarily focuses on three core aspects: lactate production and transport, lactyltransferases (Writers), and delactylases (Erasers).

Lactate dehydrogenase (LDH) and monocarboxylate transporters (MCT) are critical targets for regulating lactate levels (Hatami et al., 2023). LDH inhibitors, such as FX11 and Gossypol, significantly lower the intracellular lactate levels and lactylation by blocking the conversion of pyruvate to lactate, thereby inhibiting tumor proliferation and metastasis. This effect has been validated in prostate cancer and triple-negative breast cancer models (Xian et al., 2015; Mazzio et al., 2021). Meanwhile, MCT1 inhibitor AZD3965 prevents lactate efflux, reducing lactate accumulation in the tumor microenvironment (TME), showing antitumor activity in preclinical models of lung and breast cancer (Beloueche-Babari et al., 2017).

Lactyltransferases (Writers) and delactylases (Erasers) are direct regulators of lactylation. p300 and KAT8 are prominent targets in current research. Inhibitors of p300, such as A-485 and C646, block lactylation on both histones and non-histone proteins, suppressing the transcription of tumor-related genes (Yang et al., 2013; Ji et al., 2022). These inhibitors exhibit significant antitumor effects in prostate cancer and triple-negative breast cancer models (Yang et al., 2013; Ji et al., 2022). KAT8 is an emerging target, and its inhibitors are expected to further expand the regulatory scope of lactylation (Xie et al., 2024). Conversely, SIRT3 acts as a delactylase by removing lactylation modifications through an NAD^+^-dependent mechanism (Jin et al., 2023). Activators of SIRT3, such as Nicotinamide Riboside (NR) and Nicotinamide Mononucleotide (NMN), enhance SIRT3 activity, decrease lactylation levels, and inhibit liver cancer progression while sensitizing chemotherapy (Mukherjee et al., 2021; Zhao et al., 2022b).

In terms of cancer immunotherapy, lactylation remodels the immune microenvironment (TME) to promote immune evasion (Chen et al., 2025). Lactate-driven lactylation induces regulatory T cell (Treg) differentiation, promotes tumor-associated macrophage (M2-type TAM) polarization, and upregulates PD-L1 expression, inhibiting the activity of CD8^+^ T cells and NK cells (Chen et al., 2025). Targeting lactylation offers new strategies to reverse immune suppression. For instance, LDH inhibitor or MCT inhibitor AZD3965 reduces lactate levels in the TME, promoting the transition of TAMs to a pro-inflammatory M1 phenotype and enhancing T cell cytotoxicity (Chen et al., 2025; Kes et al., 2020). p300 inhibitor A-485 downregulates PD-L1 expression, reducing immune checkpoint-mediated inhibition (Liu et al., 2020).

Lactylation exhibits complex regulatory roles in the cGAS-STING, TLR, and RIG-I signaling pathways. Although the specific modification sites of lactylation in the cGAS-STING, RIG and TLR pathways have not been comprehensively elucidated, it has been reported that lactylation can influences the signaling transmission of these critical immune pathways by altering protein conformation, stability, and protein-protein interactions (Li et al., 2024a; Zhang et al., 2024c; Irizarry-Caro et al., 2020; Gu et al., 2024c; Liu and Ge, 2024; Wang et al., 2024d).

## 6 Challenges and personalized treatment strategies for targeting lactylation

Lactylation, a post-translational modification driven by lactate, plays a crucial role in tumor metabolism and immune regulation, but its levels exhibit significant heterogeneity across different tumor subtypes and patients. This heterogeneity is primarily influenced by tumor metabolic patterns, genetic mutations, and the TME (Chen et al., 2025). First, tumor metabolic patterns significantly influence lactylation levels (Sui et al., 2025). Tumors with high glycolysis exhibit higher lactylation levels due to increased lactate accumulation, which suppresses anti-tumor gene expression through epigenetic silencing and directly inhibits cGAS activity, blocking STING-dependent IFN-I signaling. Second, genetic mutations play a critical role in regulating lactylation levels. For instance, KRAS mutations enhance glycolysis to increase lactylation levels leading to reduced IFN-β production (Liu et al., 2023c). Conversely, IDH1/2 mutations decrease lactylation levels through metabolic competition, indirectly restoring STING pathway activity. Additionally, the immune cell composition of TME influences lactylation levels (Wang et al., 2021). Tumor-associated macrophages (TAMs) promote lactylation by secreting lactate, inhibiting dendritic cells (DCs) from activating STING and secreting IFN-I, while CD8^+^ T cells counteract lactylation through IFN-γ secretion, maintaining immune-metabolic balance (Chen et al., 2025). However, the precise impact of targeting lactylation on cGAS-STING, TLR, and RIG-I signaling pathways remains unclear and requires further investigation.

Despite the promising potential of targeting lactylation in cancer therapy, multiple challenges remain, including heterogeneity and bidirectional regulation, biomarker development and application, and single-cell multi-omics research. Although lactylation inhibits the cGAS-STING pathway, it may enhance IFN signaling through other pathways, leading to therapeutic contradictions. Therefore, it is of great significance to develop spatiotemporal-specific regulatory strategies (such as designing nanomedicines that target tumor cell lactylation) and preserve immune cell STING activity in the future. Additionally, exploring dynamic biomarkers for lactylation and interferon signaling interactions can help accurately identify patient populations likely to benefit. Integrating single-cell metabolomics with innate immune response profiling may elucidate the immune-metabolic network underlying lactylation heterogeneity, paving the way for personalized cancer therapy.

## References

[B1] Alburquerque-Bejar J. J. Navajas-Chocarro P. Saigi M. Ferrero-Andres A. Morillas J. M. Vilarrubi A. (2023). MYC activation impairs cell-intrinsic IFNγ signaling and confers resistance to anti-PD1/PD-L1 therapy in lung cancer. Cell Rep. Med. 4 (4), 101006. 10.1016/j.xcrm.2023.101006 37044092 PMC10140599

[B2] An R. Wu C. Tang C. Zhang C. Han F. Xu Z. (2024). Blockade of CD73 potentiates radiotherapy antitumor immunity and abscopal effects via STING pathway. Cell Death Discov. 10 (1), 404. 10.1038/s41420-024-02171-4 39285178 PMC11405876

[B3] Apostolova P. Pearce E. L. (2022). Lactic acid and lactate: revisiting the physiological roles in the tumor microenvironment. Trends Immunol. 43 (12), 969–977. 10.1016/j.it.2022.10.005 36319537 PMC10905416

[B50] Author Anonymous (2022). IRF3 and IRF7 require SIRT1 for liquid-liquid phase separation and transactivation of IFN-1. Nat. Immunol. 23 (8), 1144–1145. 10.1038/s41590-022-01270-7 35879453 PMC9310678

[B4] Bade B. C. Dela Cruz C. S. (2020). Lung cancer 2020: epidemiology, etiology, and prevention. Clin. Chest Med. 41 (1), 1–24. 10.1016/j.ccm.2019.10.001 32008623

[B5] Baglivo S. Bianconi F. Metro G. Gili A. Tofanetti F. R. Bellezza G. (2021). Higher TLR7 gene expression predicts poor clinical outcome in advanced NSCLC patients treated with immunotherapy. Genes (Basel) 12 (7), 992. 10.3390/genes12070992 34209514 PMC8303258

[B6] Bao Y. Pan Z. Zhao L. Qiu J. Cheng J. Liu L. (2024). BIBR1532 combined with radiotherapy induces ferroptosis in NSCLC cells and activates cGAS-STING pathway to promote anti-tumor immunity. J. Transl. Med. 22 (1), 519. 10.1186/s12967-024-05331-3 38816831 PMC11138045

[B7] Beloueche-Babari M. Wantuch S. Casals Galobart T. Koniordou M. Parkes H. G. Arunan V. (2017). MCT1 inhibitor AZD3965 increases mitochondrial metabolism, facilitating combination therapy and noninvasive magnetic resonance spectroscopy. Cancer Res. 77 (21), 5913–5924. 10.1158/0008-5472.CAN-16-2686 28923861 PMC5669455

[B8] Bragelmann J. Lorenz C. Borchmann S. Nishii K. Wegner J. Meder L. (2021). MAPK-pathway inhibition mediates inflammatory reprogramming and sensitizes tumors to targeted activation of innate immunity sensor RIG-I. Nat. Commun. 12 (1), 5505. 10.1038/s41467-021-25728-8 34535668 PMC8448826

[B9] Byun J. K. (2023). Tumor lactic acid: a potential target for cancer therapy. Arch. Pharm. Res. 46 (2), 90–110. 10.1007/s12272-023-01431-8 36729274

[B10] Chakraborty S. Ye J. Wang H. Sun M. Zhang Y. Sang X. (2023). Application of toll-like receptors (TLRs) and their agonists in cancer vaccines and immunotherapy. Front. Immunol. 14, 1227833. 10.3389/fimmu.2023.1227833 37936697 PMC10626551

[B11] Chang X. Lu T. Xu R. Wang C. Zhao J. Zhang L. (2022). Identification of lactate metabolism-related subtypes and development of a lactate-related prognostic indicator of lung adenocarcinoma. Front. Genet. 13, 949310. 10.3389/fgene.2022.949310 36092870 PMC9449370

[B12] Chen J. Huang Z. Chen Y. Tian H. Chai P. Shen Y. (2025). Lactate and lactylation in cancer. Signal Transduct. Target Ther. 10 (1), 38. 10.1038/s41392-024-02082-x 39934144 PMC11814237

[B13] Chen J. Zhao D. Wang Y. Liu M. Zhang Y. Feng T. (2024). Lactylated apolipoprotein C-ii induces immunotherapy resistance by promoting extracellular lipolysis. Adv. Sci. (Weinh) 11 (38), e2406333. 10.1002/advs.202406333 38981044 PMC11481198

[B14] Chen L. Huang L. Gu Y. Cang W. Sun P. Xiang Y. (2022b). Lactate-lactylation hands between metabolic reprogramming and immunosuppression. Int. J. Mol. Sci. 23 (19), 11943. 10.3390/ijms231911943 36233246 PMC9569569

[B15] Chen Q. Wang Y. Yang L. Sun L. Wen Y. Huang Y. (2022a). PM2.5 promotes NSCLC carcinogenesis through translationally and transcriptionally activating DLAT-mediated glycolysis reprograming. J. Exp. Clin. Cancer Res. 41 (1), 229. 10.1186/s13046-022-02437-8 35869499 PMC9308224

[B16] Chen S. Zhou X. Yang X. Li W. Li S. Hu Z. (2021). Dual blockade of lactate/GPR81 and PD-1/PD-L1 pathways enhances the anti-tumor effects of metformin. Biomolecules 11 (9), 1373. 10.3390/biom11091373 34572586 PMC8466555

[B17] Cheng G. Wu J. Ji M. Hu W. Wu C. Jiang J. (2023). TET2 inhibits the proliferation and metastasis of lung adenocarcinoma cells via activation of the cGAS-STING signalling pathway. BMC Cancer 23 (1), 825. 10.1186/s12885-023-11343-x 37667220 PMC10478367

[B18] Chin E. N. Sulpizio A. Lairson L. L. (2023). Targeting STING to promote antitumor immunity. Trends Cell Biol. 33 (3), 189–203. 10.1016/j.tcb.2022.06.010 35931610

[B20] Demaria O. Cornen S. Daeron M. Morel Y. Medzhitov R. Vivier E. (2019). Harnessing innate immunity in cancer therapy. Nature 574 (7776), 45–56. 10.1038/s41586-019-1593-5 31578484

[B21] de Sousa V. M. L. Carvalho L. (2018). Heterogeneity in lung cancer. Pathobiology 85 (1-2), 96–107. 10.1159/000487440 29635240

[B22] Dong H. He X. Zhang L. Chen W. Lin Y. C. Liu S. B. (2024). Targeting PRMT9-mediated arginine methylation suppresses cancer stem cell maintenance and elicits cGAS-mediated anticancer immunity. Nat. Cancer 5 (4), 601–624. 10.1038/s43018-024-00736-x 38413714 PMC11056319

[B23] Donzelli J. Proestler E. Riedel A. Nevermann S. Hertel B. Guenther A. (2021). Small extracellular vesicle-derived miR-574-5p regulates PGE2-biosynthesis via TLR7/8 in lung cancer. J. Extracell. Vesicles 10 (12), e12143. 10.1002/jev2.12143 34596365 PMC8485338

[B24] Duan W. Liu W. Xia S. Zhou Y. Tang M. Xu M. (2023). Warburg effect enhanced by AKR1B10 promotes acquired resistance to pemetrexed in lung cancer-derived brain metastasis. J. Transl. Med. 21 (1), 547. 10.1186/s12967-023-04403-0 37587486 PMC10428599

[B25] Exposito F. Redrado M. Houry M. Hastings K. Molero-Abraham M. Lozano T. (2023). PTEN loss confers resistance to anti-PD-1 therapy in non-small cell lung cancer by increasing tumor infiltration of regulatory T cells. Cancer Res. 83 (15), 2513–2526. 10.1158/0008-5472.CAN-22-3023 37311042

[B26] Fan H. Yang F. Xiao Z. Luo H. Chen H. Chen Z. (2023b). Lactylation: novel epigenetic regulatory and therapeutic opportunities. Am. J. Physiol. Endocrinol. Metab. 324 (4), E330–E338. 10.1152/ajpendo.00159.2022 36856188

[B27] Fan Z. Liu Z. Zhang N. Wei W. Cheng K. Sun H. (2023a). Identification of SIRT3 as an eraser of H4K16la. iScience 26 (10), 107757. 10.1016/j.isci.2023.107757 37720100 PMC10504495

[B28] Farhat K. Riekenberg S. Heine H. Debarry J. Lang R. Mages J. (2008). Heterodimerization of TLR2 with TLR1 or TLR6 expands the ligand spectrum but does not lead to differential signaling. J. Leukoc. Biol. 83 (3), 692–701. 10.1189/jlb.0807586 18056480

[B29] Feng F. Wu J. Chi Q. Wang S. Liu W. Yang L. (2024). Lactylome analysis unveils lactylation-dependent mechanisms of stemness remodeling in the liver cancer stem cells. Adv. Sci. (Weinh) 11 (38), e2405975. 10.1002/advs.202405975 39099416 PMC11481176

[B30] Fu H. Y. Li C. Yang W. Gai X. D. Jia T. Lei Y. M. (2013). FOXP3 and TLR4 protein expression are correlated in non-small cell lung cancer: implications for tumor progression and escape. Acta histochem. 115 (2), 151–157. 10.1016/j.acthis.2012.06.002 22749378

[B31] Gaffney D. O. Jennings E. Q. Anderson C. C. Marentette J. O. Shi T. Schou Oxvig A. M. (2020). Non-enzymatic lysine lactoylation of glycolytic enzymes. Cell Chem. Biol. 27 (2), 206–213. 10.1016/j.chembiol.2019.11.005 31767537 PMC7395678

[B32] Gallinari P. Di Marco S. Jones P. Pallaoro M. Steinkuhler C. (2007). HDACs, histone deacetylation and gene transcription: from molecular biology to cancer therapeutics. Cell Res. 17 (3), 195–211. 10.1038/sj.cr.7310149 17325692

[B33] Garcia-Martinez L. Zhang Y. Nakata Y. Chan H. L. Morey L. (2021). Epigenetic mechanisms in breast cancer therapy and resistance. Nat. Commun. 12 (1), 1786. 10.1038/s41467-021-22024-3 33741974 PMC7979820

[B34] Gergen A. K. Kohtz P. D. Halpern A. L. Li A. Meng X. Reece T. B. (2020). Activation of toll-like receptor 2 promotes proliferation of human lung adenocarcinoma cells. Anticancer Res. 40 (10), 5361–5369. 10.21873/anticanres.14544 32988855

[B35] Gu C. Wang X. Wang K. Xie F. Chen L. Ji H. (2024b). Cryoablation triggers type I interferon-dependent antitumor immunity and potentiates immunotherapy efficacy in lung cancer. J. Immunother. Cancer 12 (1), e008386. 10.1136/jitc-2023-008386 38272564 PMC10824009

[B36] Gu J. Xu X. Li X. Yue L. Zhu X. Chen Q. (2024c). Tumor-resident microbiota contributes to colorectal cancer liver metastasis by lactylation and immune modulation. Oncogene 43 (31), 2389–2404. 10.1038/s41388-024-03080-7 38890429 PMC11281901

[B37] Gu X. Zhu Y. Su J. Wang S. Su X. Ding X. (2024a). Lactate-induced activation of tumor-associated fibroblasts and IL-8-mediated macrophage recruitment promote lung cancer progression. Redox Biol. 74, 103209. 10.1016/j.redox.2024.103209 38861833 PMC11215341

[B38] Guo Z. Tang Y. Wang S. Huang Y. Chi Q. Xu K. (2024). Natural product fargesin interferes with H3 histone lactylation via targeting PKM2 to inhibit non-small cell lung cancer tumorigenesis. Biofactors 50 (3), 592–607. 10.1002/biof.2031 38149461

[B39] Handy D. E. Castro R. Loscalzo J. (2011). Epigenetic modifications: basic mechanisms and role in cardiovascular disease. Circulation 123 (19), 2145–2156. 10.1161/CIRCULATIONAHA.110.956839 21576679 PMC3107542

[B40] Hatami H. Sajedi A. Mir S. M. Memar M. Y. (2023). Importance of lactate dehydrogenase (LDH) and monocarboxylate transporters (MCTs) in cancer cells. Health Sci. Rep. 6 (1), e996. 10.1002/hsr2.996 36570342 PMC9768844

[B41] He Y. Jiang S. Zhong Y. Wang X. Cui Y. Liang J. (2023). USP7 promotes non-small-cell lung cancer cell glycolysis and survival by stabilizing and activating c-Abl. Clin. Transl. Med. 13 (12), e1509. 10.1002/ctm2.1509 38082439 PMC10713873

[B42] Hirsch F. R. Scagliotti G. V. Mulshine J. L. Kwon R. Curran W. J. Jr. Wu Y. L. (2017). Lung cancer: current therapies and new targeted treatments. Lancet 389 (10066), 299–311. 10.1016/S0140-6736(16)30958-8 27574741

[B43] Hoden B. DeRubeis D. Martinez-Moczygemba M. Ramos K. S. Zhang D. (2022). Understanding the role of Toll-like receptors in lung cancer immunity and immunotherapy. Front. Immunol. 13, 1033483. 10.3389/fimmu.2022.1033483 36389785 PMC9659925

[B44] Holicek P. Truxova I. Rakova J. Salek C. Hensler M. Kovar M. (2023). Type I interferon signaling in malignant blasts contributes to treatment efficacy in AML patients. Cell Death Dis. 14 (3), 209. 10.1038/s41419-023-05728-w 36964168 PMC10039058

[B45] Hopfner K. P. Hornung V. (2020). Molecular mechanisms and cellular functions of cGAS-STING signalling. Nat. Rev. Mol. Cell Biol. 21 (9), 501–521. 10.1038/s41580-020-0244-x 32424334

[B46] Hu M. Zhao Y. Cao Y. Tang Q. Feng Z. Ni J. (2020). DRP1 promotes lactate utilization in KRAS-mutant non-small-cell lung cancer cells. Cancer Sci. 111 (10), 3588–3599. 10.1111/cas.14603 32767829 PMC7540982

[B47] Hu X. Huang X. Yang Y. Sun Y. Zhao Y. Zhang Z. (2024b). Dux activates metabolism-lactylation-MET network during early iPSC reprogramming with Brg1 as the histone lactylation reader. Nucleic Acids Res. 52 (10), 5529–5548. 10.1093/nar/gkae183 38512058 PMC11162783

[B48] Hu Y. He Z. Li Z. Wang Y. Wu N. Sun H. (2024a). Lactylation: the novel histone modification influence on gene expression, protein function, and disease. Clin. Epigenetics 16 (1), 72. 10.1186/s13148-024-01682-2 38812044 PMC11138093

[B49] Huang H. Tang S. Ji M. Tang Z. Shimada M. Liu X. (2018). p300-Mediated lysine 2-hydroxyisobutyrylation regulates glycolysis. Mol. Cell 70 (4), 663–678. 10.1016/j.molcel.2018.04.011 29775581 PMC6029451

[B51] Irizarry-Caro R. A. McDaniel M. M. Overcast G. R. Jain V. G. Troutman T. D. Pasare C. (2020). TLR signaling adapter BCAP regulates inflammatory to reparatory macrophage transition by promoting histone lactylation. Proc. Natl. Acad. Sci. U. S. A. 117 (48), 30628–30638. 10.1073/pnas.2009778117 33199625 PMC7720107

[B52] Ivashkiv L. B. Donlin L. T. (2014). Regulation of type I interferon responses. Nat. Rev. Immunol. 14 (1), 36–49. 10.1038/nri3581 24362405 PMC4084561

[B53] Jacoberger-Foissac C. Cousineau I. Bareche Y. Allard D. Chrobak P. Allard B. (2023). CD73 inhibits cGAS-STING and cooperates with CD39 to promote pancreatic cancer. Cancer Immunol. Res. 11 (1), 56–71. 10.1158/2326-6066.CIR-22-0260 36409930 PMC9812927

[B54] Ji C. Xu W. Ding H. Chen Z. Shi C. Han J. (2022). The p300 inhibitor A-485 exerts antitumor activity in growth hormone pituitary adenoma. J. Clin. Endocrinol. Metab. 107 (6), e2291–e2300. 10.1210/clinem/dgac128 35247260 PMC9113810

[B55] Jiang J. Huang D. Jiang Y. Hou J. Tian M. Li J. (2021). Lactate modulates cellular metabolism through histone lactylation-mediated gene expression in non-small cell lung cancer. Front. Oncol. 11, 647559. 10.3389/fonc.2021.647559 34150616 PMC8208031

[B56] Jiang Y. Zhang H. Wang J. Chen J. Guo Z. Liu Y. (2023). Exploiting RIG-I-like receptor pathway for cancer immunotherapy. J. Hematol. Oncol. 16 (1), 8. 10.1186/s13045-023-01405-9 36755342 PMC9906624

[B57] Jin J. Bai L. Wang D. Ding W. Cao Z. Yan P. (2023). SIRT3-dependent delactylation of cyclin E2 prevents hepatocellular carcinoma growth. EMBO Rep. 24 (5), e56052. 10.15252/embr.202256052 36896611 PMC10157311

[B58] Ka N. L. Park M. K. Kim S. S. Jeon Y. Hwang S. Kim S. M. (2023). NR1D1 stimulates antitumor immune responses in breast cancer by activating cGAS-STING signaling. Cancer Res. 83 (18), 3045–3058. 10.1158/0008-5472.CAN-23-0329 37395684 PMC10538367

[B59] Kang X. Li P. Zhang C. Zhao Y. Hu H. Wen G. (2020). The TLR4/ERK/PD-L1 axis may contribute to NSCLC initiation. Int. J. Oncol. 57 (2), 456–465. 10.3892/ijo.2020.5068 32468028 PMC7307593

[B60] Kes M. M. G. Van den Bossche J. Griffioen A. W. Huijbers E. J. M. (2020). Oncometabolites lactate and succinate drive pro-angiogenic macrophage response in tumors. Biochim. Biophys. Acta Rev. Cancer 1874 (2), 188427. 10.1016/j.bbcan.2020.188427 32961257

[B61] Kim H. Kim H. Feng Y. Li Y. Tamiya H. Tocci S. (2020). PRMT5 control of cGAS/STING and NLRC5 pathways defines melanoma response to antitumor immunity. Sci. Transl. Med. 12 (551), eaaz5683. 10.1126/scitranslmed.aaz5683 32641491 PMC7508354

[B62] Kim M. J. Kim J. Y. Shin J. H. Kang Y. Lee J. S. Son J. (2023). FFAR2 antagonizes TLR2-and TLR3-induced lung cancer progression via the inhibition of AMPK-TAK1 signaling axis for the activation of NF-κB. Cell Biosci. 13 (1), 102. 10.1186/s13578-023-01038-y 37287005 PMC10249240

[B63] Li B. Zhu L. Lu C. Wang C. Wang H. Jin H. (2021). circNDUFB2 inhibits non-small cell lung cancer progression via destabilizing IGF2BPs and activating anti-tumor immunity. Nat. Commun. 12 (1), 295. 10.1038/s41467-020-20527-z 33436560 PMC7804955

[B64] Li C. Choi H. P. Wang X. Wu F. Chen X. Lu X. (2017). Post-translational modification of human histone by wide tolerance of acetylation. Cells 6 (4), 34. 10.3390/cells6040034 29023412 PMC5753069

[B65] Li H. Liu C. Li R. Zhou L. Ran Y. Yang Q. (2024a). AARS1 and AARS2 sense L-lactate to regulate cGAS as global lysine lactyltransferases. Nature 634 (8036), 1229–1237. 10.1038/s41586-024-07992-y 39322678

[B66] Li J. Wu Z. Chen G. Wang X. Zhu X. Zhang Y. (2023). Formosanin C inhibits non-small-cell lung cancer progression by blocking MCT4/CD147-mediated lactate export. Phytomedicine 109, 154618. 10.1016/j.phymed.2022.154618 36610137

[B67] Li W. Zhou C. Yu L. Hou Z. Liu H. Kong L. (2024b). Tumor-derived lactate promotes resistance to bevacizumab treatment by facilitating autophagy enhancer protein RUBCNL expression through histone H3 lysine 18 lactylation (H3K18la) in colorectal cancer. Autophagy 20 (1), 114–130. 10.1080/15548627.2023.2249762 37615625 PMC10761097

[B68] Li X. Chen M. Chen X. He X. Li X. Wei H. (2024c). TRAP1 drives smooth muscle cell senescence and promotes atherosclerosis via HDAC3-primed histone H4 lysine 12 lactylation. Eur. Heart J. 45 (39), 4219–4235. 10.1093/eurheartj/ehae379 39088352 PMC11481199

[B69] Li X. Yang Y. Zhang B. Lin X. Fu X. An Y. (2022b). Lactate metabolism in human health and disease. Signal Transduct. Target Ther. 7 (1), 305. 10.1038/s41392-022-01151-3 36050306 PMC9434547

[B70] Li Y. Gao Y. Jiang X. Cheng Y. Zhang J. Xu L. (2022a). SAMHD1 silencing cooperates with radiotherapy to enhance anti-tumor immunity through IFI16-STING pathway in lung adenocarcinoma. J. Transl. Med. 20 (1), 628. 10.1186/s12967-022-03844-3 36578072 PMC9798699

[B71] Lim K. H. Staudt L. M. (2013). Toll-like receptor signaling. Cold Spring Harb. Perspect. Biol. 5 (1), a011247. 10.1101/cshperspect.a011247 23284045 PMC3579400

[B72] Liu B. Zhang J. Meng X. Xie S. M. Liu F. Chen H. (2023b). HDAC6-G3BP2 promotes lysosomal-TSC2 and suppresses mTORC1 under ETV4 targeting-induced low-lactate stress in non-small cell lung cancer. Oncogene 42 (15), 1181–1195. 10.1038/s41388-023-02641-6 36823378

[B73] Liu H. Ge B. (2024). Lactylation as a post-translational regulator of cGAS and immunity. Mol. Cell 84 (23), 4483–4485. 10.1016/j.molcel.2024.11.018 39642855

[B74] Liu J. He D. Cheng L. Huang C. Zhang Y. Rao X. (2020). p300/CBP inhibition enhances the efficacy of programmed death-ligand 1 blockade treatment in prostate cancer. Oncogene 39 (19), 3939–3951. 10.1038/s41388-020-1270-z 32203167 PMC7210073

[B75] Liu J. van der Hoeven R. Kattan W. E. Chang J. T. Montufar-Solis D. Chen W. (2023c). Glycolysis regulates KRAS plasma membrane localization and function through defined glycosphingolipids. Nat. Commun. 14 (1), 465. 10.1038/s41467-023-36128-5 36709325 PMC9884228

[B76] Liu X. Qin H. Zhang L. Jia C. Chao Z. Qin X. (2023a). Hyperoxia induces glucose metabolism reprogramming and intracellular acidification by suppressing MYC/MCT1 axis in lung cancer. Redox Biol. 61, 102647. 10.1016/j.redox.2023.102647 36867943 PMC10011425

[B77] Liu Y. Wang F. Peng D. Zhang D. Liu L. Wei J. (2024). Activation and antitumor immunity of CD8(+) T cells are supported by the glucose transporter GLUT10 and disrupted by lactic acid. Sci. Transl. Med. 16 (762), eadk7399. 10.1126/scitranslmed.adk7399 39196962

[B78] Long Y. Guo J. Chen J. Sun J. Wang H. Peng X. (2023). GPR162 activates STING dependent DNA damage pathway as a novel tumor suppressor and radiation sensitizer. Signal Transduct. Target Ther. 8 (1), 48. 10.1038/s41392-022-01224-3 36725837 PMC9892510

[B79] Luo Z. Li Y. Xu B. Yu T. Luo M. You P. (2024). Overexpression of ESYT3 improves radioimmune responses through activating cGAS-STING pathway in lung adenocarcinoma. Exp. Hematol. Oncol. 13 (1), 77. 10.1186/s40164-024-00546-y 39103908 PMC11302107

[B80] Lv M. Chen M. Zhang R. Zhang W. Wang C. Zhang Y. (2020). Manganese is critical for antitumor immune responses via cGAS-STING and improves the efficacy of clinical immunotherapy. Cell Res. 30 (11), 966–979. 10.1038/s41422-020-00395-4 32839553 PMC7785004

[B81] Lv X. Lv Y. Dai X. (2023). Lactate, histone lactylation and cancer hallmarks. Expert Rev. Mol. Med. 25, e7. 10.1017/erm.2022.42 36621008

[B82] Ma B. Akosman B. Kamle S. Lee C. M. He C. H. Koo J. S. (2021). CHI3L1 regulates PD-L1 and anti-CHI3L1-PD-1 antibody elicits synergistic antitumor responses. J. Clin. Invest 131 (21), e137750. 10.1172/JCI137750 34720089 PMC8553560

[B83] Ma F. Lei Y. Y. Ding M. G. Luo L. H. Xie Y. C. Liu X. L. (2020). LncRNA NEAT1 interacted with DNMT1 to regulate malignant phenotype of cancer cell and cytotoxic T cell infiltration via epigenetic inhibition of p53, cGAS, and STING in lung cancer. Front. Genet. 11, 250. 10.3389/fgene.2020.00250 32296457 PMC7136539

[B84] Marmorstein R. Zhou M. M. (2014). Writers and readers of histone acetylation: structure, mechanism, and inhibition. Cold Spring Harb. Perspect. Biol. 6 (7), a018762. 10.1101/cshperspect.a018762 24984779 PMC4067988

[B85] Marzio A. Kurz E. Sahni J. M. Di Feo G. Puccini J. Jiang S. (2022). EMSY inhibits homologous recombination repair and the interferon response, promoting lung cancer immune evasion. Cell 185 (1), 169–183.e19. 10.1016/j.cell.2021.12.005 34963055 PMC8751279

[B86] Mazzio E. Mack N. Badisa R. B. Soliman K. F. A. (2021). Triple isozyme lactic acid dehydrogenase inhibition in fully viable MDA-MB-231 cells induces cytostatic effects that are not reversed by exogenous lactic acid. Biomolecules 11 (12), 1751. 10.3390/biom11121751 34944395 PMC8698706

[B87] Miller J. L. Grant P. A. (2013). The role of DNA methylation and histone modifications in transcriptional regulation in humans. Subcell. Biochem. 61, 289–317. 10.1007/978-94-007-4525-4_13 23150256 PMC6611551

[B88] Moreno-Yruela C. Zhang D. Wei W. Baek M. Liu W. Gao J. (2022). Class I histone deacetylases (HDAC1-3) are histone lysine delactylases. Sci. Adv. 8 (3), eabi6696. 10.1126/sciadv.abi6696 35044827 PMC8769552

[B89] Mukherjee S. Mo J. Paolella L. M. Perry C. E. Toth J. Hugo M. M. (2021). SIRT3 is required for liver regeneration but not for the beneficial effect of nicotinamide riboside. JCI Insight 6 (7), e147193. 10.1172/jci.insight.147193 33690226 PMC8119200

[B90] Niu Z. Chen C. Wang S. Lu C. Wu Z. Wang A. (2024). HBO1 catalyzes lysine lactylation and mediates histone H3K9la to regulate gene transcription. Nat. Commun. 15 (1), 3561. 10.1038/s41467-024-47900-6 38670996 PMC11053077

[B91] Nooreldeen R. Bach H. (2021). Current and future development in lung cancer diagnosis. Int. J. Mol. Sci. 22 (16), 8661. 10.3390/ijms22168661 34445366 PMC8395394

[B92] Pan R. Y. He L. Zhang J. Liu X. Liao Y. Gao J. (2022). Positive feedback regulation of microglial glucose metabolism by histone H4 lysine 12 lactylation in Alzheimer's disease. Cell Metab. 34 (4), 634–648 e6. 10.1016/j.cmet.2022.02.013 35303422

[B93] Pang Y. Zhou Y. Wang Y. Fang L. Xiao S. (2024). Lactate-lactylation-HSPA6 axis promotes PRRSV replication by impairing IFN-beta production. J. Virol. 98 (1), e0167023. 10.1128/jvi.01670-23 38088561 PMC10804950

[B94] Perry J. L. Tian S. Sengottuvel N. Harrison E. B. Gorentla B. K. Kapadia C. H. (2020). Pulmonary delivery of nanoparticle-bound toll-like receptor 9 agonist for the treatment of metastatic lung cancer. ACS Nano 14 (6), 7200–7215. 10.1021/acsnano.0c02207 32463690 PMC7531260

[B95] Qian Y. Galan-Cobo A. Guijarro I. Dang M. Molkentine D. Poteete A. (2023). MCT4-dependent lactate secretion suppresses antitumor immunity in LKB1-deficient lung adenocarcinoma. Cancer Cell 41 (7), 1363–1380 e7. 10.1016/j.ccell.2023.05.015 37327788 PMC11161201

[B96] Qiu J. Xia Y. Bao Y. Cheng J. Liu L. Qian D. (2024). Silencing PinX1 enhances radiosensitivity and antitumor-immunity of radiotherapy in non-small cell lung cancer. J. Transl. Med. 22 (1), 228. 10.1186/s12967-024-05023-y 38431575 PMC10908107

[B97] Sato Y. Yoshino H. Tsuruga E. Kashiwakura I. (2020). Fas ligand enhances apoptosis of human lung cancer cells cotreated with RIG-I-like receptor agonist and radiation. Curr. Cancer Drug Targets 20 (5), 372–381. 10.2174/1568009620666200115161717 31951181

[B98] Schneegans S. Loptien J. Mojzisch A. Loreth D. Kretz O. Raschdorf C. (2024). HERC5 downregulation in non-small cell lung cancer is associated with altered energy metabolism and metastasis. J. Exp. Clin. Cancer Res. 43 (1), 110. 10.1186/s13046-024-03020-z 38605423 PMC11008035

[B99] Schweibenz B. D. Solotchi M. Hanpude P. Devarkar S. C. Patel S. S. (2023). RIG-I recognizes metabolite-capped RNAs as signaling ligands. Nucleic Acids Res. 51 (15), 8102–8114. 10.1093/nar/gkad518 37326006 PMC10450190

[B100] Shang S. Wang M. Z. Xing Z. He N. Li S. (2022). Lactate regulators contribute to tumor microenvironment and predict prognosis in lung adenocarcinoma. Front. Immunol. 13, 1024925. 10.3389/fimmu.2022.1024925 36505423 PMC9732022

[B101] Sui Y. Shen Z. Wang Z. Feng J. Zhou G. (2025). Lactylation in cancer: metabolic mechanism and therapeutic strategies. Cell Death Discov. 11 (1), 68. 10.1038/s41420-025-02349-4 39979245 PMC11842571

[B102] Sun D. Qian H. Wang J. Xie T. Teng F. Li J. (2022). ARID1A deficiency reverses the response to anti-PD(L)1 therapy in EGFR-mutant lung adenocarcinoma by enhancing autophagy-inhibited type I interferon production. Cell Commun. Signal 20 (1), 156. 10.1186/s12964-022-00958-5 36229854 PMC9558404

[B103] Sun J. Wang X. Liu W. Ji P. Shang A. Wu J. (2020). Novel evidence for retinoic acid-induced G (Rig-G) as a tumor suppressor by activating p53 signaling pathway in lung cancer. FASEB J. 34 (9), 11900–11912. 10.1096/fj.201903220R 32741018 PMC7725982

[B104] Sun M. Bai Y. Zhao S. Liu X. Gao Y. Wang L. (2018). Gram-negative bacteria facilitate tumor progression through TLR4/IL-33 pathway in patients with non-small-cell lung cancer. Oncotarget 9 (17), 13462–13473. 10.18632/oncotarget.24008 29568370 PMC5862591

[B105] Taguchi T. Mukai K. (2019). Innate immunity signalling and membrane trafficking. Curr. Opin. Cell Biol. 59, 1–7. 10.1016/j.ceb.2019.02.002 30875551

[B106] Tan Y. Zhu Q. Yang M. Yang F. Zeng Q. Jiang Z. (2024). Tetrandrine activates STING/TBK1/IRF3 pathway to potentiate anti-PD-1 immunotherapy efficacy in non-small cell lung cancer. Pharmacol. Res. 207, 107314. 10.1016/j.phrs.2024.107314 39059614

[B107] Taniguchi H. Caeser R. Chavan S. S. Zhan Y. A. Chow A. Manoj P. (2022). WEE1 inhibition enhances the antitumor immune response to PD-L1 blockade by the concomitant activation of STING and STAT1 pathways in SCLC. Cell Rep. 39 (7), 110814. 10.1016/j.celrep.2022.110814 35584676 PMC9449677

[B108] Taniguchi H. Chakraborty S. Takahashi N. Banerjee A. Caeser R. Zhan Y. A. (2024). ATR inhibition activates cancer cell cGAS/STING-interferon signaling and promotes antitumor immunity in small-cell lung cancer. Sci. Adv. 10 (39), eado4618. 10.1126/sciadv.ado4618 39331709 PMC11430494

[B109] Thoresen D. Wang W. Galls D. Guo R. Xu L. Pyle A. M. (2021). The molecular mechanism of RIG-I activation and signaling. Immunol. Rev. 304 (1), 154–168. 10.1111/imr.13022 34514601 PMC9293153

[B110] Trono P. Tocci A. Palermo B. Di Carlo A. D'Ambrosio L. D'Andrea D. (2023). hMENA isoforms regulate cancer intrinsic type I IFN signaling and extrinsic mechanisms of resistance to immune checkpoint blockade in NSCLC. J. Immunother. Cancer 11 (8), e006913. 10.1136/jitc-2023-006913 37612043 PMC10450042

[B111] Velasco W. V. Khosravi N. Castro-Pando S. Torres-Garza N. Grimaldo M. T. Krishna A. (2023). Toll-like receptors 2, 4, and 9 modulate promoting effect of COPD-like airway inflammation on K-ras-driven lung cancer through activation of the MyD88/NF-ĸB pathway in the airway epithelium. Front. Immunol. 14, 1118721. 10.3389/fimmu.2023.1118721 37283745 PMC10240392

[B112] Vichaya E. G. Ford B. G. Quave C. B. Rishi M. R. Grossberg A. J. Dantzer R. (2020). Toll-like receptor 4 mediates the development of fatigue in the murine Lewis Lung Carcinoma model independently of activation of macrophages and microglia. Psychoneuroendocrinology 122, 104874. 10.1016/j.psyneuen.2020.104874 32979744 PMC7686070

[B113] Vinod N. Hwang D. Azam S. H. Van Swearingen A. E. D. Wayne E. Fussell S. C. (2020). High-capacity poly(2-oxazoline) formulation of TLR 7/8 agonist extends survival in a chemo-insensitive, metastatic model of lung adenocarcinoma. Sci. Adv. 6 (25), eaba5542. 10.1126/sciadv.aba5542 32596460 PMC7299629

[B114] Vlachostergios P. J. Oikonomou K. G. Gibilaro E. Apergis G. (2015). Elevated lactic acid is a negative prognostic factor in metastatic lung cancer. Cancer Biomark. 15 (6), 725–734. 10.3233/CBM-150514 26406401 PMC12965472

[B115] Wang J. Yang P. Yu T. Gao M. Liu D. Zhang J. (2022b). Lactylation of PKM2 suppresses inflammatory metabolic adaptation in pro-inflammatory macrophages. Int. J. Biol. Sci. 18 (16), 6210–6225. 10.7150/ijbs.75434 36439872 PMC9682528

[B116] Wang J. X. Choi S. Y. C. Niu X. Kang N. Xue H. Killam J. (2020b). Lactic acid and an acidic tumor microenvironment suppress anticancer immunity. Int. J. Mol. Sci. 21 (21), 8363. 10.3390/ijms21218363 33171818 PMC7664620

[B117] Wang K. Wang J. Wei F. Zhao N. Yang F. Ren X. (2017). Expression of TLR4 in non-small cell lung cancer is associated with PD-L1 and poor prognosis in patients receiving pulmonectomy. Front. Immunol. 8, 456. 10.3389/fimmu.2017.00456 28484456 PMC5399072

[B118] Wang L. Cai Z. Gu Q. Xu C. (2024d). cGAS deficiency regulates the phenotypic polarization and glycolysis of microglia through lactylation in hypoxic-ischemic encephalopathy cell model. Biochem. Genet. 62 (5), 3961–3976. 10.1007/s10528-023-10631-2 38246965

[B119] Wang L. Ma Y. Zhang S. Yang Y. Huang B. (2024b). NFATc2 promotes lactate and M2 macrophage polarization through USP17 in lung adenocarcinoma. Anticancer Drugs 35 (5), 385–396. 10.1097/CAD.0000000000001582 38386130

[B120] Wang M. He T. Meng D. Lv W. Ye J. Cheng L. (2023a). BZW2 modulates lung adenocarcinoma progression through glycolysis-mediated IDH3G lactylation modification. J. Proteome Res. 22 (12), 3854–3865. 10.1021/acs.jproteome.3c00518 37955350

[B121] Wang N. Wang W. Wang X. Mang G. Chen J. Yan X. (2022a). Histone lactylation boosts reparative gene activation post-myocardial infarction. Circ. Res. 131 (11), 893–908. 10.1161/CIRCRESAHA.122.320488 36268709

[B122] Wang P. Xie D. Xiao T. Cheng C. Wang D. Sun J. (2024a). H3K18 lactylation promotes the progression of arsenite-related idiopathic pulmonary fibrosis via YTHDF1/m6A/NREP. J. Hazard Mater 461, 132582. 10.1016/j.jhazmat.2023.132582 37742376

[B123] Wang Y. Cheng X. Liu X. Xu J. Wang L. Zhang S. (2024c). Design and synthesis of 3-(2H-Chromen-3-yl)-5-aryl-1,2,4-oxadiazole derivatives as novel toll-like receptor 2/1 agonists that inhibit lung cancer *in vitro* and *in vivo* . J. Med. Chem. 67 (6), 4583–4602. 10.1021/acs.jmedchem.3c01984 38498304

[B124] Wang Y. Meng L. Meng S. Huang L. Luo S. Wu X. (2023b). Flotillin-1 enhances radioresistance through reducing radiation-induced DNA damage and promoting immune escape via STING signaling pathway in non-small cell lung cancer. Cancer Biol. Ther. 24 (1), 2203332. 10.1080/15384047.2023.2203332 37131290 PMC10158545

[B125] Wang Y. Zou S. Zhao Z. Liu P. Ke C. Xu S. (2020a). New insights into small-cell lung cancer development and therapy. Cell Biol. Int. 44 (8), 1564–1576. 10.1002/cbin.11359 32281704 PMC7496722

[B126] Wang Z. Lu C. Zhang K. Lin C. Wu F. Tang X. (2022c). Metformin combining PD-1 inhibitor enhanced anti-tumor efficacy in STK11 mutant lung cancer through AXIN-1-dependent inhibition of STING ubiquitination. Front. Mol. Biosci. 9, 780200. 10.3389/fmolb.2022.780200 35281267 PMC8905189

[B127] Wang Z. H. Peng W. B. Zhang P. Yang X. P. Zhou Q. (2021). Lactate in the tumour microenvironment: from immune modulation to therapy. EBioMedicine 73, 103627. 10.1016/j.ebiom.2021.103627 34656878 PMC8524104

[B128] Wei L. Yang X. Wang J. Wang Z. Wang Q. Ding Y. (2023). H3K18 lactylation of senescent microglia potentiates brain aging and Alzheimer's disease through the NFκB signaling pathway. J. Neuroinflammation 20 (1), 208. 10.1186/s12974-023-02879-7 37697347 PMC10494370

[B129] Welander C. E. (1987). Overview of preclinical and clinical studies of interferon alfa-2b in combination with cytotoxic drugs. Invest New Drugs 5 (Suppl. l), S47–S59. 10.1007/BF00207263 3298133

[B130] Wu M. Zhang J. B. Xiong Y. W. Zhao Y. X. Zheng M. G. Huang X. L. (2023). Promotion of lung cancer metastasis by SIRT2-mediated extracellular protein deacetylation. Adv. Sci. (Weinh) 10 (3), e2205462. 10.1002/advs.202205462 36453571 PMC9875677

[B131] Xian Z. Y. Liu J. M. Chen Q. K. Chen H. Z. Ye C. J. Xue J. (2015). Inhibition of LDHA suppresses tumor progression in prostate cancer. Tumour Biol. 36 (10), 8093–8100. 10.1007/s13277-015-3540-x 25983002 PMC4605959

[B132] Xie B. Zhang M. Li J. Cui J. Zhang P. Liu F. (2024). KAT8-catalyzed lactylation promotes eEF1A2-mediated protein synthesis and colorectal carcinogenesis. Proc. Natl. Acad. Sci. U. S. A. 121 (8), e2314128121. 10.1073/pnas.2314128121 38359291 PMC10895275

[B133] Xie Y. Hu H. Liu M. Zhou T. Cheng X. Huang W. (2022). The role and mechanism of histone lactylation in health and diseases. Front. Genet. 13, 949252. 10.3389/fgene.2022.949252 36081996 PMC9445422

[B134] Xu K. Zhang K. Wang Y. Gu Y. (2024). Comprehensive review of histone lactylation: structure, function, and therapeutic targets. Biochem. Pharmacol. 225, 116331. 10.1016/j.bcp.2024.116331 38821374

[B135] Xu X. X. Wan H. Nie L. Shao T. Xiang L. X. Shao J. Z. (2018). RIG-I: a multifunctional protein beyond a pattern recognition receptor. Protein Cell 9 (3), 246–253. 10.1007/s13238-017-0431-5 28593618 PMC5829270

[B136] Xu Y. Meng W. Dai Y. Xu L. Ding N. Zhang J. (2025). Anaerobic metabolism promotes breast cancer survival via Histone-3 Lysine-18 lactylation mediating PPARD axis. Cell Death Discov. 11 (1), 54. 10.1038/s41420-025-02334-x 39922804 PMC11807217

[B137] Yan F. Teng Y. Li X. Zhong Y. Li C. Yan F. (2024). Hypoxia promotes non-small cell lung cancer cell stemness, migration, and invasion via promoting glycolysis by lactylation of SOX9. Cancer Biol. Ther. 25 (1), 2304161. 10.1080/15384047.2024.2304161 38226837 PMC10793688

[B138] Yan X. Yao C. Fang C. Han M. Gong C. Hu D. (2022). Rocaglamide promotes the infiltration and antitumor immunity of NK cells by activating cGAS-STING signaling in non-small cell lung cancer. Int. J. Biol. Sci. 18 (2), 585–598. 10.7150/ijbs.65019 35002511 PMC8741839

[B139] Yang H. Pinello C. E. Luo J. Li D. Wang Y. Zhao L. Y. (2013). Small-molecule inhibitors of acetyltransferase p300 identified by high-throughput screening are potent anticancer agents. Mol. Cancer Ther. 12 (5), 610–620. 10.1158/1535-7163.MCT-12-0930 23625935 PMC3651759

[B140] Yang K. Fan M. Wang X. Xu J. Wang Y. Tu F. (2022). Lactate promotes macrophage HMGB1 lactylation, acetylation, and exosomal release in polymicrobial sepsis. Cell Death Differ. 29 (1), 133–146. 10.1038/s41418-021-00841-9 34363018 PMC8738735

[B141] Yi M. Li T. Niu M. Mei Q. Zhao B. Chu Q. (2023). Exploiting innate immunity for cancer immunotherapy. Mol. Cancer 22 (1), 187. 10.1186/s12943-023-01885-w 38008741 PMC10680233

[B142] Yoshida R. Saigi M. Tani T. Springer B. F. Shibata H. Kitajima S. (2022). MET-induced CD73 restrains STING-mediated immunogenicity of EGFR-mutant lung cancer. Cancer Res. 82 (21), 4079–4092. 10.1158/0008-5472.CAN-22-0770 36066413 PMC9627131

[B143] Yu J. Chai P. Xie M. Ge S. Ruan J. Fan X. (2021). Histone lactylation drives oncogenesis by facilitating m(6)A reader protein YTHDF2 expression in ocular melanoma. Genome Biol. 22 (1), 85. 10.1186/s13059-021-02308-z 33726814 PMC7962360

[B144] Zeng Q. Wang K. Zhao Y. Ma Q. Chen Z. Huang W. (2023). Effects of the acetyltransferase p300 on tumour regulation from the novel perspective of posttranslational protein modification. Biomolecules 13 (3), 417. 10.3390/biom13030417 36979352 PMC10046601

[B145] Zhang C. Zhou L. Zhang M. Du Y. Li C. Ren H. (2024a). H3K18 lactylation potentiates immune escape of non-small cell lung cancer. Cancer Res. 84 (21), 3589–3601. 10.1158/0008-5472.CAN-23-3513 39137401

[B146] Zhang D. Tang Z. Huang H. Zhou G. Cui C. Weng Y. (2019). Metabolic regulation of gene expression by histone lactylation. Nature 574 (7779), 575–580. 10.1038/s41586-019-1678-1 31645732 PMC6818755

[B147] Zhang J. Ji H. Liu M. Zheng M. Wen Z. Shen H. (2024c). Mitochondrial DNA programs lactylation of cGAS to induce IFN responses in patients with systemic lupus erythematosus. J. Immunol. 213 (6), 795–807. 10.4049/jimmunol.2300758 39093026

[B148] Zhang R. Li L. Yu J. (2024b). Lactate-induced IGF1R protein lactylation promotes proliferation and metabolic reprogramming of lung cancer cells. Open Life Sci. 19 (1), 20220874. 10.1515/biol-2022-0874 38840891 PMC11151389

[B149] Zhang X. Bai X. C. Chen Z. J. (2020). Structures and mechanisms in the cGAS-STING innate immunity pathway. Immunity 53 (1), 43–53. 10.1016/j.immuni.2020.05.013 32668227

[B150] Zhang Z. Kuo J. C. Yao S. Zhang C. Khan H. Lee R. J. (2021). CpG oligodeoxynucleotides for anticancer monotherapy from preclinical stages to clinical trials. Pharmaceutics 14 (1), 73. 10.3390/pharmaceutics14010073 35056969 PMC8780291

[B151] Zhao L. Yuan H. Wang Y. Geng Y. Yun H. Zheng W. (2023). HBV confers innate immune evasion through triggering HAT1/acetylation of H4K5/H4K12/miR-181a-5p or KPNA2/cGAS-STING/IFN-I signaling. J. Med. Virol. 95 (7), e28966. 10.1002/jmv.28966 37466313

[B152] Zhao Q. Zhou J. Li F. Guo S. Zhang L. Li J. (2022b). The role and therapeutic perspectives of sirtuin 3 in cancer metabolism reprogramming, metastasis, and chemoresistance. Front. Oncol. 12, 910963. 10.3389/fonc.2022.910963 35832551 PMC9272524

[B153] Zhao X. Hu S. Zeng L. Liu X. Song Y. Zhang Y. (2022a). Irradiation combined with PD-L1(-/-) and autophagy inhibition enhances the antitumor effect of lung cancer via cGAS-STING-mediated T cell activation. iScience 25 (8), 104690. 10.1016/j.isci.2022.104690 35847556 PMC9283938

[B154] Zheng J. Shi W. Yang Z. Chen J. Qi A. Yang Y. (2023). RIG-I-like receptors: molecular mechanism of activation and signaling. Adv. Immunol. 158, 1–74. 10.1016/bs.ai.2023.03.001 37453753

[B155] Zhou J. Zhang L. Liu S. DeRubeis D. Zhang D. (2024). Toll-like receptors in breast cancer immunity and immunotherapy. Front. Immunol. 15, 1418025. 10.3389/fimmu.2024.1418025 38903515 PMC11187004

